# Genetic Risk Factors for Essential Tremor: A Review

**DOI:** 10.5334/tohm.67

**Published:** 2020-06-11

**Authors:** Vasileios Siokas, Athina-Maria Aloizou, Zisis Tsouris, Ioannis Liampas, Paraskevi Aslanidou, Metaxia Dastamani, Alexandros G. Brotis, Dimitrios P. Bogdanos, Georgios M. Hadjigeorgiou, Efthimios Dardiotis

**Affiliations:** 1 Department of Neurology, Laboratory of Neurogenetics, University Hospital of Larissa, Greece, Faculty of Medicine, School of Health Sciences, University of Thessaly, Larissa, GR; 2 Department of Neurosurgery, School of Medicine, University Hospital of Larissa, University of Thessaly, Larissa, GR; 3 Department of Rheumatology and Clinical Immunology, University General Hospital of Larissa, Faculty of Medicine, School of Health Sciences, University of Thessaly, Viopolis 40500, Larissa, GR; 4 Department of Neurology, Medical School, University of Cyprus, Nicosia, CY

**Keywords:** essential tremor, genetic polymorphism, single nucleotide polymorphism, variant, tremor, hyperkinetic movements, movement disorders

## Abstract

**Highlights:**

In the current review, we thoroughly reviewed 74 identified articles regarding genes and genetic loci that confer susceptibility to ET. Over 50 genes/genetic loci have been examined for possible association with ET, but consistent results failed to be reported raising the need for collaborative multiethnic studies.

**Background:**

Essential tremor (ET) is a common movement disorder, which is mainly characterized by bilateral tremor (postural and/or kinetic) in the upper limbs, with other parts of the body possibly involved. While the pathophysiology of ET is still unclear, there is accumulating evidence indicating that genetic variability may be heavily involved in ET pathogenesis. This review focuses on the role of genetic risk factors in ET susceptibility.

**Methods:**

The PubMed database was searched for articles written in English, for studies with humans with ET, controls without ET, and genetic variants. The terms “essential tremor” and “polymorphism” (as free words) were used during search. We also performed meta-analyses for the most examined genetic variants.

**Results:**

Seventy four articles concerning *LINGO1, LINGO2, LINGO4, SLC1A2, STK32B, PPARGC1A, CTNNA3, DRD3, ALAD, VDR, HMOX1, HMOX2, LRRK1,LRRK2, GBA, SNCA, MAPT, FUS, CYPsIL17A, IL1B, NOS1, ADH1B, TREM2, RIT2, HNMT, MTHFR, PPP2R2B, GSTP1, PON1, GABA receptors* and *GABA transporter, HS1BP3, ADH2, hSKCa3* and *CACNL1A4* genes, and ETM genetic loci were included in the current review. Results from meta-analyses revealed a marginal association for the STK32B rs10937625 and a marginal trend for association (in sensitivity analysis) for the LINGO1 rs9652490, with ET.

**Discussion:**

Quite a few variants have been examined for their possible association with ET. LINGO1 rs9652490 and STK32B rs10937625 appear to influence, to some extent, ET susceptibility. However, the conflicting results and the lack of replication for many candidate genes raise the need for collaborative multiethnic studies.

## Introduction

Essential tremor (ET) is a common movement disorder, which is mainly characterized by bilateral tremor (postural and/or kinetic) in the upper limbs, but can also spread to other parts of the body (e.g. jaw, head) [1,2]. Phenotypically, ET is a tremor manifesting during voluntary movements, with a frequency of 4–12-Hz, and usually manifests as mildly asymmetric [3]. Alongside the motor manifestations, non-motor symptoms (e.g. REM-sleep behavior disorder, cognitive dysfunction, sensory abnormalities, dysautonomic symptoms, depression) may also be present [4,5,6,7,8].

ET compromises the commonest movement disorder of adulthood, while its onset may span from childhood to late ages [9]. Few environmental factors have been implicated as possible risk factors for ET [10,11,12,13]. Thus, the consumption of b-carboline alkaloid, caffeine and ethanol, harmane, exposure to pesticides, lead and other heavy metals, are all considered as potential risk factors for ET [10,11,13]. On the other hand, antioxidants and smoking may protect against ET [10,11,13]. However, the most widely established risk factors for ET are considered the family history of ET and aging [3,12].

The exact pathophysiological processes that lead to ET are still poorly understood [14]. Despite the possible contribution of specific environmental factors in ET development, also genetic factors probably contribute to ET risk. The significance of genes to ET risk has been demonstrated via the identification of genetic variants from familial studies [15,16], studies in twins [17], and the emerged variants derived from candidate gene association studies (CGASs) [18] and genome wide association studies (GWASs) [19,20,21].

In this review article we discuss the available scientific data regarding the role of genetics in ET, by giving emphasis to the results from CGASs. Moreover, we discuss the main findings from GWASs. We also performed meta-analyses for the most examined genetic variants. Our aim is to shed some light on which variants may predispose to ET.

## Methods -Study identification and selection

**Figure 1:**
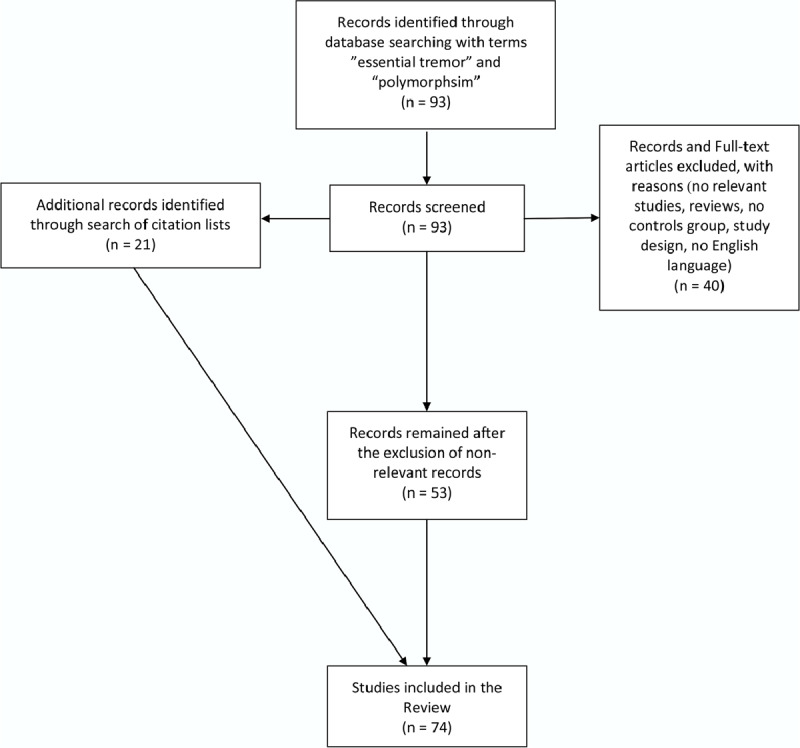
Flow chart presenting the selection of the studies included in the current review.

PubMed was searched for eligible studies written in English. We searched for articles from the inception of PubMed up to July 2019, for studies in humans, regarding ET and genetic variants. The terms used were “essential tremor” and “polymorphisms” as free words. The complete search algorithm can be accessed in the **Supporting File 1**. PubMed was searched for the last time on July 20^th^, 2019. Additionally, reference lists of retrieved articles were scanned for supplementary eligible articles. The flowchart with the selection process of the included studies is depicted in Figure 1. We included published articles between 1997 and 2019.

From each study, we extracted the following data when possible: 1) author, 2) year of publication, 3) ethnicity of the population, 4) numbers of cases and controls, 5) age at disease onset, 6) mean age of examination, 7) sex distribution, 8) genotyped variants, 9) family history of the participants, 10) diagnosis assessment, 11) correction for multiple comparisons, and 12) assessment of Hardy-Weinberg Equilibrium (HWE).

For the *LINGO1* rs9652490, *LINGO1* rs11856808, *SLC1A2* rs3794087, *STK32B* rs10937625 and *PPARGC1A* rs17590046 we performed meta-analyses. We included data from CGASs. Data from GWASs (neither from discovery nor from follow-up phases) were not included. The heterogeneity was calculated using the Cochran’s Q and I^2^ index. In case of high heterogeneity (P_Q_ < 0.10 and/or I^2^ > 75%), the random-effects model [22,23,24] was applied. Otherwise, the fixed-effects model [25] was used. Publication bias was estimated with Egger’s test [26] when possible, with a p < 0.10 as suggestive of publication bias. The magnitude of association was calculated for the allelic model using the Review Manager (RevMan) Version 5.3 software (http://tech.cochrane.org/revman), with p < 0.05 as the statistically significant threshold. Allele counts were recalculated from data given as percentages, if necessary. For the analysis for LINGO1 rs9652490 and the studies of Vilarino-Guel et al. [27,28], we included data only from the larger study [28], in order to avoid possible overlap. We also conducted a sensitivity analysis for LINGO1 rs9652490 by omitting one study at a time to examine the effect of each individual study.

## Results and Discussion

### Literature Review

Seventy four studies published between 1997 and 2019 were included in the current review. Baseline characteristics from the studies regarding *LINGO1, LINGO2, LINGO4, SLC1A2, STK32B, PPARGC1A, CTNNA3, DRD3, ALAD, VDR, HMOX1, HMOX2, LRRK1, LRRK2, GBA, SNCA, MAPT, FUS, CYPs, IL17A, IL1B, NOS1, ADH1B, TREM2, RIT2, HNMT, MTHFR, PPP2R2B, GSTP1, PON1, GABA receptors* and *GABA transporter, HS1BP3, ADH2, hSKCa3* and *CACNL1A4* genes, and ETM genetic loci, are accessible in **Supporting File 2**. Gene, chromosome position, possible mechanism of function, total number of studies (with comparison between ET cases and Controls), number of studies with association, number of studies without association and sample characteristics for the most examined genes (*LINGO1, DRD3, SLC1A2, LRRK2, FUS/TLS, SNCA, MAPT, HMOX1, HMOX2*) for association with ET, are presented at Table 1.

**Table 1:** Gene, chromosome position, possible mechanisms of function, total number of studies (with comparison between ET cases and Controls), number of studies with association, number of studies without association and sample characteristics for the most examined genes for association with ET (Data from GWASs not included).

Gene	Chromosome position^*1^	Possible Mechanisms	Number of studies			Studies with association				Studies without association			

			Total	With association	Without association	In Caucasians/North Americans	In Asians	Total Cases	Total Controls	In Caucasians/North Americans	In Asians	Total Cases	Total Controls

LINGO1	Chromosome 15: 77,613,027–77,820,900	neurite outgrowth, axonal regeneration, regulation of the myelination and neuronal survival	11	7	4	5	2	2,751	3,073	2	2	711	2,186
DRD3	Chromosome 3: 114,128,652–114,199,407	dopamine receptor	8	3	5	3	0	507	516	4	1	919	1,266
SLC1A2	Chromosome 11: 35,251,205–35,420,063	regulates glutamate at synaptic cleft and extracellularly	5	2	3	0	2	630	2,204	2	1	749	1,657
LRRK2	Chromosome 12: 40,196,744–40,369,285	elevated LRRK2 kinase activity leads to neuronal toxicity	5	1	4	0	1^*2^	450	827	1	3	1,165	3,650
FUS/TLS	Chromosome 16: 31,180,110–31,194,871	ALS and FTD pathways	5*^3^	1	4	0	1	513	6,169	3	1	729	1,251
SNCA	Chromosome 4: 89,724,099–89,838,315	PD pathways	3	1	2	1	0	46	100	2	0	767	1,406
MAPT	Chromosome 17: 45,894,382–46,028,334	PD pathways	3	1	2	1	0	356	409	2	0	449	528
HMOX1	Chromosome 22: 35,380,361–35,394,207	heme catabolism, lead toxicity	3	1	2	1	0	202	747	1	1	427	447
HMOX2	Chromosome 16: 4,474,690–4,510,347	heme catabolism, lead toxicity	3	2	1*^4^	2	0	404	965	0	1	225	229

ET, essential tremor; LINGO1, leucine-rich repeat and lg domain containing nogo receptor-interacting protein 1; SLC1A2, solute carrier family 1 member 2; LRRK2, leucine-rich repeat kinase-2; FUS/TLS, Fused in Sarcoma/Translocated in Liposarcoma; DRD3, dopamine D3 receptor; SNCA, a-synuclein; MAPT, microtubule associated protein tau; HMOX1, Heme Oxygenase 1; HMOX2, Heme Oxygenase 2; ALS, amyotrophic lateral sclerosis; FTD, frontotemporal dementia; PD, Parkinson’s disease; GWASs, genome-wide association studies.

*1 Based on https://www.ensembl.org/index.html.

*2 90% Asians.

*3 Rajput et al. (2014) and Merner et al. (2012) are not included.

*4 The interaction of ALAD rs1800435 with the HMOX2 rs1051308 could be weakly associated with the familial ET.

#### 1. LINGO (LINGO 1, LINGO 2 and LINGO 4) genes

##### 1.1. LINGO 1 (Leucine rich repeat and Immunoglobulin-like domain-containing protein 1)

LINGO1 is thought to be implicated in neurite outgrowth, axonal regeneration, regulation of the myelination and neuronal survival [19], while its inactivation seems to protect from degeneration and enhance the survival of the neurons [3,29,30]. Published data indicate that defective function of LINGO1 may lead to Purkinje cell loss and axonal dysfunction, and therefore, possibly to ET [19,31,32].

The rs9652490 and the rs11856808, located in intron 3 across the *LINGO1* gene, were the two first variants that emerged as potential risk factors for ET through the first GWAS conducted in patients with ET [19]. In greater detail, the G allele of the rs9652490 was associated with ET in the initial discovery analysis of an Icelandic population [Odds Ratio (OR) = 1.63, p = 3 × 10^–7^], and in the combined sample of follow-up, which consisted of Austrian, German, American and Icelandic datasets. Most importantly, it reached the genome-wide significant association threshold in both of the analyses, in the discovery and the follow-up data-sets. The association of the rs9652490 and ET was further replicated in a few CGASs, in North American Caucasians [27,28] [for the major allele (p = 0.014, OR = 2.2) [27] and (p = 0.026, OR = 0.63 for recessive model) [28]], North Americans (Non-Hispanic whites) (in patients with ‘definite’ or ‘probable’ ET (p = 0.03, OR = 1.41) [31], Asian (p = 0.00036, OR = 2.59) [33], German (p = 0.009, OR = 1.61) [20] and French (p = 0.046, OR = 1.70) [20] samples. However, the positive results were not reproduced in Spanish [34], Chinese [35,36], Asian [37], Latvian [38] and French-Canadian [39] populations.

The other variant that emerged as a possible risk factor for ET through the GWAS from Stefansson et al., was the T allele of the rs11856808. It was associated with ET in the initial discovery analysis of the Icelandic population (OR = 1.51, p = 3 × 10^–6^), but in the follow-up sample this association with ET was not revealed after adjustment for the rs965249 [19]. Overall, the results from the CGASs, following this GWAS, failed to replicate these results suggesting that rs11856808 is not a major genetic risk factor for ET [31,34,35,38,39], as it was found to confer susceptibility to ET only in German and French populations [20].

Apart from rs9652490 and rs11856808, some other *LINGO1* variants have also been associated with ET. Rs2271397 (p = 0.017, OR = 2.139), ss491228439 (p = 0.038, OR = 1.812) and the A465-C474-C714 haplotype (p = 0.041, OR = 1.8) were associated with increased ET risk in females of a Chinese Han population [36]. Rs7177008, rs13313467 and rs8028808, were associated with early-onset ET (p = 0.028, OR = 1.52; p = 0.0238, OR = 1.54; and p = 0.0391, OR = 1.55, respectively) in North Americans (Non-Hispanic whites) [31], while rs4886887 (OR = 1.83, p = 0.018 for recessive model), rs3144 (OR = 1.48, p = 0.03 for recessive model), rs8028808 (OR = 0.49, p = 0.008 for recessive model), and rs12905478 (OR = 0.36 p = 0.02) were associated with ET, with rs907396 influencing age at onset of ET (p = 0.019), in North America Caucasians [28]. Finally, rs8030859 was associated with ET in Germans (OR = 1.72, p = 0.00105) [40].

##### 1.2. LINGO 2 (Leucine rich repeat and Immunoglobulin-like domain-containing protein 2)

The LINGO2 protein presents high homology (over 50%) to LINGO1, but constitutes a much less characterized paralog [28,41]. Despite the unknown function of LINGO2, it is considered to share some functions with LINGO1 and was therefore a target in ET CGASs. However, based on studies in mice, LINGO2 appears to be restricted to neuronal tissue [42], a feature that may differentiate it from the LINGO3 and the LINGO4 paralogs [28].

Two studies so far have examined the effect of *LINGO2* in ET, concerning a few variants [28,41]. Of these, rs10812774 and rs7033345 have been shown to influence the age at onset of ET, as carriers of these variants appear to have an earlier age at onset by 4 to 5 years in a study on Caucasians from North America [28], while they were also associated with ET (OR = 1.50, p = 0.04 for rs7033345 and OR = 1.56, p = 0.01 for rs10812774 in recessive model) in Asians from Singapore [41]. Moreover, the rs1412229 has been associated with ET (OR = 0.72, p = 0.015 in recessive model) [28].

##### 1.3. LINGO 4 (Leucine rich repeat and Immunoglobulin-like domain-containing protein 4)

The LINGO4 protein is another paralog of the LINGO1 protein, with an amino acid resemblance to LINGO of almost 50% [43]. One study has so far examined the role of two variants [the T>A transition (rs61746299), driving the amino acid change Thr444Ser, and the C>T transition (rs1521179), located 12 bp downstream to the end of coding region) across *LINGO4* gene variants in Chinese Han patients from Mainland China, failing to reach statistical significance, though [44].

#### 2. SLC1A2 (Solute carrier family 1 – glial affinity glutamate transporter-member 2)

The solute carrier family 1 – glial affinity glutamate transporter-member 2 (SLC1A2) gene, [also known as Excitatory amino acid transporter 2 (EATT2) or glutamate transporter 1 (GLT-1)], encodes SLC1A2, a member of the group of solute transporter proteins [45]. Elevated levels of glutamate in the synaptic cleft and extracellularly are neurotoxic and have been associated with neurodegeneration. Defective function of SLC1A2 can lead to increased glutamate levels, and consequently to neurotoxicity [46]. A relation between SLC1A2 and ET can be found when one examines the pathophysiology of other factors in ET; the fact that ethanol relieves ET, while it increases the SLC1A2 expression, highlights the protein’s importance, as well the elevated expression of SLC1A2 in the inferior olive where are produced the oscillations responsible for the tremor [6,20,47,48,49].

The second GWAS exploring the genetics of ET in participants from Germany, Austria, and Denmark, reported that the rs3794087 across the *SLC1A2* was associated with ET (OR = 1.46, p = 6.95 × 10^–5^) [20]. The statistical significance was evident in both stages of the GWAS, as well in the subgroup analysis in ET patients with a ‘definite’ diagnosis [20], revealing the robustness of the results.

Since then, 5 further GCASs attempted to replicate the finding of this GWAS, in China, North America, Taiwan and Spain [50,51,52,53,54]. Based on these results, the *SLC1A2* rs3794087 A allele was more frequent in ET patients compared to controls in Taiwanese [53] and Chinese groups [51]. Based on the previous reports, it is however rather unlikely that the *SLC1A2* rs3794087 consists a major risk factor for ET.

#### 3. STK32B (serine/threonine kinase 32B), PPARGC1A (PPARG Coactivator 1 Alpha), CTNNA3 (Catenin Alpha 3)

The third GWAS (two-stage) conducted so far, exploring the genetic susceptibility of ET included 2807 patients with ET and 6441 controls of European ancestry. Two markers, rs10937625 (OR = 0.77, p = 7.36 × 10^–4^), located in the serine/threonine kinase *STK32B* gene, and rs17590046 (OR = 0.75, p = 6.81 × 10^–4^) in the transcriptional coactivator *PPARGC1A* gene, were associated with ET [21]. Moreover, three markers, namely rs12764057 (OR = 1.17, p = 1.19 × 10^–8^), rs10822974 (OR = 1.16, p = 1.65 × 10^–7^) and rs7903491 (OR = 1.10, p = 2.49 × 10^–7^), in the cell-adhesion molecule *CTNNA3* gene were found to be statistically significant in the combined analysis of both stages [21]. The C allele of rs10937625 of the *STK32B* gene was named a protective factor and the G allele of rs7903491 of the *CTNNA3* gene a risk factor for ET in Chinese [55], while the *PPARGC1A* gene was also associated with ET in Asians [56]. However, other studies failed to replicate the results for *STK32B* (rs10937625), *PPARGC1A* (rs17590046) and *CTNNA3* (rs12764057 and rs10822974) [55,56].

#### 4. DRD3 (Dopamine D3 receptor), ETM1, ETM2 and ETM3 loci

The rs6280, (also known as 312G>A and Ser9Gly) represents a non-synonymous point mutation, where serine is replaced by glycine (Ser9Gly), in position 9 of the N terminal part of the receptor. The Ser9Gly mutation affects the extracellular N-terminus of the DRD3, which does not appear to participate in receptor ligand binding [57], a possible explanation for the lack of reproducibility of positive associations between the Ser9Gly *DRD3* variant and ET.

Thers6280, was considered a candidate genetic risk factor for ET because it is mapped in chromosome 3q13, in the ETM1 locus [58], a locus that emerged through a genome wide linkage scan in Icelandic families (ETM1; OMIM: 190300) [59]. Indeed, the linkage peak markers of the ETM1 locus, namely D3S1278 and D3S1267, are located 1 and 10 Mb far from the *DRD3* gene, respectively [59]. Another reason for the *DRD3* appropriateness as a candidate genetic risk factor for ET, was the fact that *DRD3* has been previously associated with tardive dyskinesia, phenotypic appearance of Parkinson’s Disease (PD), and with its expression reported decreased in patients with PD [60,61,62,63].

Following this train of thought, in 2006, Jeanneteau et al., reported that rs6280 was associated with risk and age at onset of ET in a Caucasian population [63] while positive associations were also found in a French sample [57]. The latest case-control study involved a Spanish population, where rs6280 was associated with risk and age at onset of ET, as well as with the risk for voice tremor [58]. Despite the robustness of the results even after a pooled meta-analysis, the magnitude of the association remained weak, suggesting that the association between rs6280 and ET may represent a false positive observation [58], and that rs6280 does not represent a strong risk factor for ET. The latter could partly explain the lack of replication of the association between this marker and ET in Latvian, Russian, Belarusians, Ukrainians, Polish, Lithuanians [64], Asians [65], Italians [66], Germans, Danish, French [67], and overall Caucasians [68].

Apart from the ETM1 locus, ETM2 (OMIM: 602134) [69] and ETM3 (OMIM: 611456) [70] have also been considered as causal genetic factors for ET in a genome wide linkage scan, without though, as in case of ETM1 locus, the genes and the causal mutations for both, ETM2 and ETM3, loci being identified [3]. Inashkina et al. performed CGAS genotyping short tandem repeat (STR) markers located within ETM1 and ETM2 loci in Latvian patients with ET, and the biggest differentiation of frequencies was found for allele 171 of the marker D2S220 (OR 0.13, 95% CI 0.02–1.03, P = 0.05) [64]. Zahorakova et al. performed a genetic analysis of three polymorphic loci (etm1231, etm1234, and etm1240) located within the ETM2 candidate region in 61 Czech patients with a family history of ET and 68 healthy controls, but the allele frequencies did not significantly differ between cases and controls [71]. Therefore, the importance of these polymorphisms is still hard to assess, as they have yet to make an appearance in other studies.

#### 5. ALAD (d-aminolevulinic acid dehydratase), VDR (Vitamin D Receptor), HMOX1 (heme oxygenase 1) and HMOX2 (heme oxygenase 2)

The d-aminolevulinic acid dehydratase (ALAD) catalyzes the second step in heme synthesis, leading to the production of cobalamin-monopyrrole-porphobilinogen [72,73], and the *ALAD* gene has been shown to influence the toxicokinetics of lead [74]. *ALAD* gene has one polymorphism, giving way to two codominant alleles, ALAD-1 and ALAD-2 [74]. The non-synonymous coding variant rs1800435 (also known as K59N and G177C), creates the ALAD-2 variant allele [72] and carriers of the ALAD-2 variant may be more susceptible to lead toxicity [74]. To sum up, the *ALAD* gene may influence heme synthesis and lead toxicity. This is of interest in the context of ET, because lead intoxication produces a syndrome involving tremor and lead has been named an environmental susceptibility factor for ET [75].

The *vitamin D receptor (VDR)* gene, encodes the vitamin D receptor, and it seems that genetic variability of *VDR* may also affect lead toxicity [73]. On the other hand, the heme oxygenase (HMOX) enzyme is also involved in heme catabolism. There are two isozymes, the inducible heme oxygenase-1 (HMOX1) and the constitutive heme oxygenase-2 (HMOX2), encoded by the *HMOX1* and *HMOX2* genes respectively [76]. As ET and PD share many features, and variants across *VDR, HMOX1*, and *HMOX2* genes have been reported to confer susceptibility to PD [77,78], and as lead exposure has been associated with PD [79], it was reasonable that variants across these genes could be targets for CGASs regarding ET.

Regarding the *ALAD* gene, the odds of ET were increased in individuals who carried an ALAD-2 allele and had an elevated blood lead concentration, when compared to individuals with only elevated blood lead concentration [74]. In a study in Caucasian Spanish, the *ALAD* rs1800435 polymorphism was not associated with familial essential tremor (FET) risk, but its interaction with the *HMOX2* rs1051308 polymorphism could be weakly associated with the FET [80]. *HMOX1* (rs2071746) and *HMOX2* (rs4786504, rs1051308) did not associate with ET in the Chinese [18], while the allelic frequencies of rs2071746T (OR = 0.76, p = 0.015) and rs1051308G (OR = 0.71, p = 0.004) were lower in Spanish white ET patients [76].

Regarding the *VDR* gene, the TT genotype of the rs2228570 was associated with sporadic essential tremor (SET) (p = 0.033; OR = 0.453 and similarly, the C allele was associated with an increased risk of SET (p = 0.033; OR = 2.207) [81], while the rs731236 did not associate with ET in Chinese [18].

#### 6. LRRK2 (Leucine-rich repeat kinase 2), LRRK1 (Leucine-rich repeat kinase 1), SNCA (non A4 component of amyloid precursor/Alpha-synuclein), GBA (Glucocerebrosidase) and MAPT (microtubule-associated protein tau)

A number of PD/parkinsonism related genes [*Leucine-rich repeat kinase 2 (LRRK2), Alpha-synuclein (SNCA), Glucocerebrosidase (GBA), microtubule-associated protein tau (MAPT)*] have been examined for possible association with ET. LRRK2 is a large multi-domain protein kinase, mainly localized in the cytoplasm [82]. Pathogenic variants of *LRRK2*, may lead to elevated LRRK2 kinase activity, which appears to mediate neuronal toxicity [82] and mutations across the *LRRK2* gene have also been incriminated for familial PD [83]. Moreover, the phenotypic appearance of PD patients with mutations on LRRK2 could be initially resembled to ET phenotype [84]. Due to all these, LRRK2 has been included as a target in ET CGASs. The *leucine-rich repeat kinase 1 (LRRK1)* gene is a paralog of LRRK2, while many variants have been linked to PD as well [85]. The *LRRK2* R1628P variant has previously been associated with ET. More precisely, carriers of R1628P appeared to have a two-fold increased risk of ET (p = 0.0035, OR = 2.20) in a cohort consisting mainly of Asians (90%) [86]. However, other examined variants of *LRRK1* and *LRRK2* genes, in Asian and Non-Hispanic whites cohorts [84,87,88,89], failed to present any association with ET.

SNCA constitutes the main component of Lewy bodies, neurites and glial cytoplasmic inclusions, which are considered as the key pathological feature in PD and multiple system atrophy (MSA) [90]. The NACP-Rep1 polymorphism is located in the promoter region of the *SNCA* gene [90,91,92]. Variants in this locus of *SNCA* have been shown to be implicated in the regulation of the *SNCA* gene expression [90] and the 263bp allele of the NACP-Rep1 was encountered more frequently in ET patients than healthy controls [90]. However, two additional studies regarding *SNCA* variability association and ET failed to provide any association [91,92].

The *GBA* gene encodes the enzyme glucocerebrosidase, which is the causative gene for Gaucher disease, a lysosomal storage disease with an autosomal recessive mode of inheritance [93]. The L444P mutation of the *GBA* gene represents the commonest pathogenic mutation for Gaucher disease in China [94]. While the N370S, R496H, E326K and the R44C mutations have been identified in ET cases [89], studies so far, regarding ET and *GBA*, have not provided evidence that the *GBA* gene is a major risk factor for ET [89,95].

Mutations in the *MAPT* gene cause frontotemporal dementia with parkinsonism linked to chromosome 17 (FTDP- 17), and additionally, the H1 haplotype of *MAPT* gene has been associated with increased risk for disorders with a-synuclein pathology [96,97,98]. Three studies have been so far conducted concerning ET and variants across the *MAPT* gene [99,100,101], but only MAPT H1 was associated with ET, among North American Caucasians [99].

#### 7. FUS/TLS (Fused in sarcoma/Translated in liposarcoma)

Pathogenic mutations in *Fused in sarcoma/Translated in liposarcoma (FUS/TLS)* are causing factors for amyotrophic lateral sclerosis (ALS) (4% of familial cases and <1% of sporadic ALS cases) [73,102] and frontotemporal lobar degeneration (FTLD) [103].

Merner et al. detected the p.Arg216Cys variant in two ET cases (one familial and one sporadic), and the p.Pro431Leu in a case with familial ET [104]. The non-pathogenic mutation p.G174_G175del in one ET patient and two healthy controls, and a novel p.R377W in one patient that had positive family history of disease, were also identified [105]. Moreover, the Met392Ile in the FUS gene has been reported to increase susceptibility to ET among a Chinese sample [106]. Ortega-Cubero et al., detected a few *FUS* gene variants in a Spanish cohort, none of them found associated with ET, however, when compared to controls from the 1000 Genomes project [107]. Finally, three other studies reported negative results [108,109,110]. Therefore, the bibliographic data lead us to believe that rare variants across the *FUS* gene may compromise a rare cause of monogenic ET [111].

#### 8. CYPs (Cytochromes P450) genes

Cytochromes P450 (CYPs) consist a large family of enzymes that oxidize steroids, fatty acids, xenobiotics, drugs, pesticides, and heavy metals, mainly aiming to the clear the organism from various compounds, while also participating in hormone synthesis and breakdown [112]. The human CYP superfamily contains over 100 functional genes and pseudogenes, while *CYP* genetic variability appears to have an effect to the risk of various diseases and to pharmacogenetics [113,114].

Primidone is a drug that appears to be effective, to some degree, in the management of ET [115]. Primidone is partly metabolized by CYP2C19 [113]. Homozygotes for the defective alleles are considered as poor metabolizers, while carriers of more functional alleles as extensive metabolizers [114]. Heterozygosis for CYP2C19*1/CYP2C19*2 has been associated with the risk for ET in Caucasians [114] and genetic alternations in *CYP2C8* and *CYP2C9* genes (which are in high genetic linkage with the *CYP2C19* gene, especially among Caucasians) [116], have further been associated with the risk for ET [114,116].

The *CYP2D6* (Cytochrome P450 2D6) gene appears to have the largest phenotypical variability among the *CYP* genes [117]. Genetic status regarding *CYP2D6* gene affects the metabolism of the debrisoquine, as carriers of defect alleles poorly metabolize debrisoquine, in contrast to the effective metabolizers, who carry functional alleles [117]. As the *CYP2D6* gene has been associated with PD, Agunez et al. genotyped 91 ET patients and 258 controls for 8 *CYP2D6* variants, but failed to detect any association [118].

#### 9. Other genes

Polymorphisms in genes associated with Restless legs syndrome (RLS) [rs8193036 of *interleukin-17A gene (IL17A)*, rs1143643, rs1143634, and rs1143633 of *interleukin-1B (IL1B)* gene, rs693534 and rs7977109 of *nitric oxide synthase 1 (NOS1)* gene and rs6413413 and rs1229984 of *alcohol dehydrogenase (ADH1B)* gene] have been examined for possible association with ET [18]. Solely rs1143633 of *IL1B* was associated with the risk of ET after adjusting for age and gender (recessive model) and after multiple comparisons correction (OR = 2.63, p = 0.002) [18].

The Triggering Receptor Expressed on Myeloid cells 2 (TREM2), coded by the *TREM2* gene [119], is a transmembrane signaling protein, pairing up with Tyrosine Kinase-binding protein (TYROBP/DP12), and is involved in innate immune system functions such as inflammation, proliferation and phagocytosis [120]. The R47H (rs75392628) is the most extensively studied genetic variant across the *TREM2* gene, which results in reduced signaling, lipoprotein uptake and binding, and surface uptake [120,121]. The rs75392628 has been associated with Alzheimer’s disease, sporadic ALS, the logopenic variant of primary progressive aphasia, and frontotemporal dementia- behavioral variant [121]. In 2015, based on the neurodegenerative hypothesis, Ortega-Cubero et al. found an association between rs75392628 and ET in a Spanish population (OR = 5.97, p = 0.042), without replication though [122].

Another variant that has been associated with ET is rs12456492 of another PD related gene, the *Ras-like without CAAX 2 (RIT2)* [123]. Additionally, the homozygosity for the missense variant (rs11558538) 105Thr genotype of the *Histamine N-Methyltransferase (HNMT)* gene was found to be more frequent in Caucasian Spanish ET patients [124], but not in Caucasian ET patients from North America [125]. The 677T, 1298C alleles, the T677T and T677T/A1298A genotypes, and the C677C/C1298C compound genotypes of *methylenetetrahydrofolate reductase (MTHFR)* gene [126], were also associated with ET [127]. Finally, the proportion of subjects carrying rare short (CAG)5–7 alleles of the *Protein Phosphatase 2 Regulatory Subunit Bbeta (PPP2R2B)* gene was higher in an ET patient cohort [4/132 (3.0%), p < 0.001] when compared to controls [1/625 [(0.2%)] [128].

Moving on, the rs1695 of the *Glutathione S-Transferase Pi 1 (GSTP1)* gene was significantly more frequently encountered in individuals with ET exposed to pesticides when compared to non-exposed patients [129]. The non-synonymous functional coding variants rs662 (Q192R) and the rs854560 (L55M) of *paraoxonase-1 (PON1)* gene [130], [which encode the homonymous serum calcium dependent esterase enzyme which mainly hydrolyze the active metabolites (oxons) of some organophosphate pesticides such as diazinon, parathion and chlorpyrifos)] [131], have not been associated with ET. Negative association studies between ET and gamma-aminobutyric acid A receptors (GABRA) [132,133,134], *gamma-aminobutyric acid receptor rho* genes [*GABRR1, GABRR2*, and *GABRR3*] [135], and *GABA transporter* genes [133], and the a A265G variant in the *HS1 binding protein 3 (HS1BP3)* gene [136], (a gene that had been previously found in families with ET [137]). Finally, there is no indication that the *alcohol dehydrogenase 2 (ADH2)* [138], the *human small conductance calcium-activated potassium channel (hSKCa3)* and the *calcium voltage-gated channel subunit alpha1 A (CACNL1A4)* genes rank among the ET genetic risk factors [139].

## Meta-analyses

**Figure 2:**
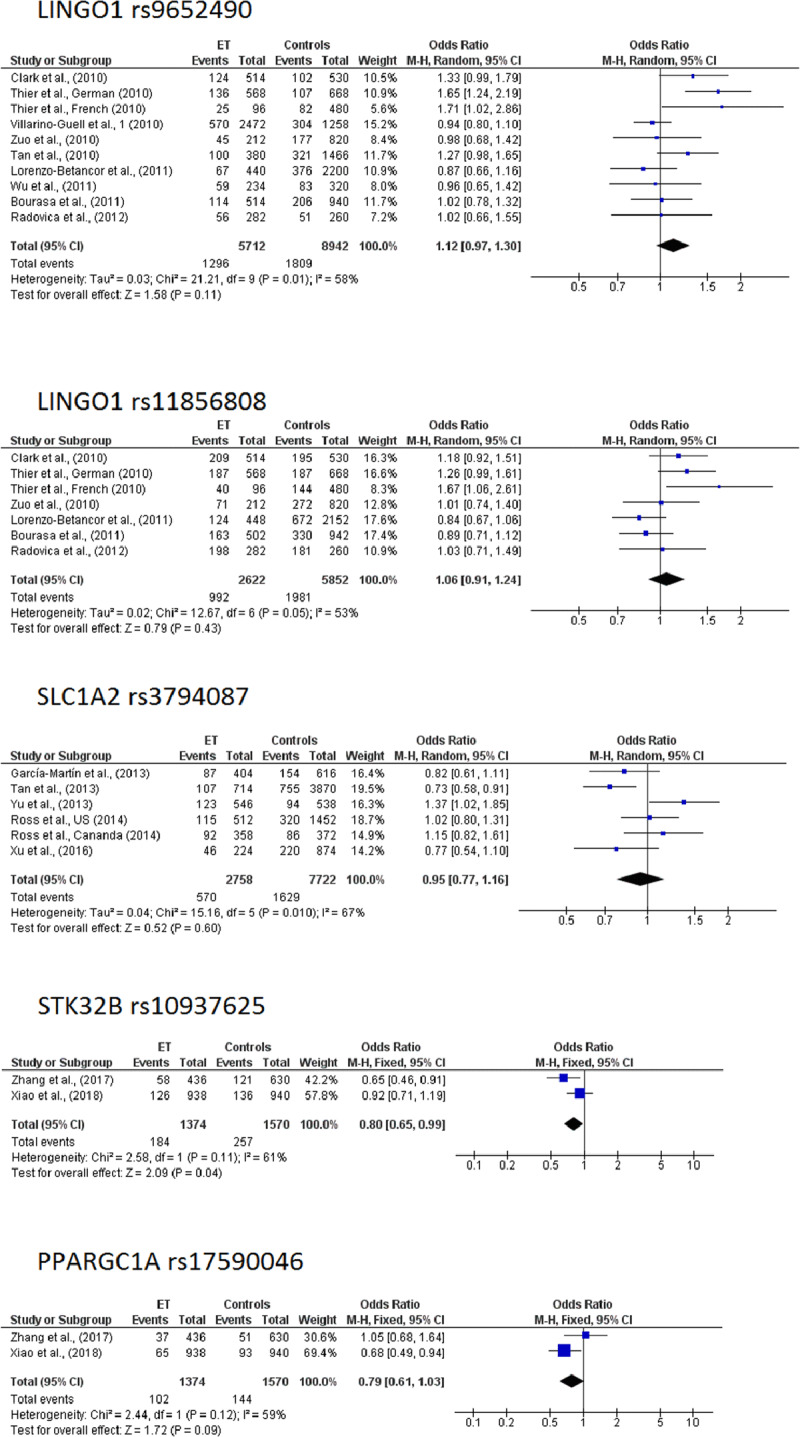
Forest Plots presenting the results from meta-analyses.

There was no indication of publication bias (p > 0.10 for Egger’s test). A marginal association was observed for the *STK32B* rs10937625 (fixed model OR: 0.80; 95%CI: 0.65–0.99, pz = 0.04) [27,28]. The results of the meta-analyses (number of included studies, I^2^, P_Q_, applied model, OR, 95% CI, p-value) regarding *LINGO1* rs9652490, *LINGO1* rs11856808, *SLC1A2* rs3794087, *STK32B* rs10937625 and *PPARGC1A* rs17590046 are summarized in Table 2. Forest plots for the overall analysis are depicted in Figure 2. In the sensitivity analysis the pooled ORs (95% CIs) ranged from 1.04 (95% CI: 0.95–1.14) to 1.16 (95% CI: 0.99–1.36). After omitting each study one at a time for the LINGO1 rs9652490, a marginal trend for association was revealed when either the study of Lorenzo-Betancor [34] (random model OR: 1.16; 95%CI: 0.99-1.35, pz = 0.06) or the one of Vilarino-Guell [28] (random model OR: 1.16; 95%CI: 0.99–1.36, pz = 0.06) was omitted. Results from sensitivity analyses are presented at Table 3, while the forest plots are accessible at **Supporting File 3**.

**Table 2:** Results from meta-analyses of the LINGO1 rs9652490, LINGO1 rs11856808, SLC1A2 rs3794087, STK32B rs10937625and PPARGC1A rs17590046 for association with ET.

Gene	Polymorphism	Number of studies^Ref.^	Population	Heterogeneity		Meta-analysis model	Test for overall effect	
	
				I^2^	P_Q_		OR (95% CI)	P-value

LINGO1	rs9652490	10 [28,31,33,34,35,37,38,39,40]	Mixed	58%	0.01	Random	1.12 (0.97–1.30)	0.11
LINGO1	rs11856808	7 [31,34,35,38,39,40]	Mixed	53%	0.05	Random	1.06 (0.91–1.24)	0.43
SLC1A2	rs3794087	6 [50,51,52,53,54]	Mixed	67%	0.01	Random	0.95 (0.77–1.16)	0.60
STK32B	rs10937625	2 [55,56]	Asian	61%	0.11	Fixed	0.80 (0.65–0.99)	0.04
PPARGC1A	rs17590046	2 [55,56]	Asian	59%	0.12	Fixed	0.79 (0.61–1.03)	0.09

ET, essential tremor; LINGO1, leucine-rich repeat and lg domain containing nogo receptor-interacting protein 1; SLC1A2, solute carrier family 1 member 2; PPARGC1A, Peroxisome Proliferator-Activated Receptor Gamma Coactivator 1-Alpha; RIT2, Ras like without CAAX 2; STK32B, serine/threonine kinase 32B; OR, odds ratio; CI, confidence interval.

**Table 3:** Results from the sensitivity meta-analyses of the LINGO1 rs9652490 for association with ET.

Omitted study	Heterogeneity		Meta-analysismodel	Test for overall effect	
	
	I^2^	P_Q_		OR (95% CI)	P-value

Clark et al., (2010) [31]	58%	0.01	Random	1.10 (0.94–1.29)	0.22
Thier et al., German (2010) [40]	33%	0.15	Fixed	1.04 (0.95–1.14)	0.43
Thier et al., French (2010) [40]	56%	0.02	Random	1.10 (0.95–1.26)	0.21
Villarino-Guell et al., 2 (2010) [28]	52%	0.04	Random	1.16 (0.99–1.36)	0.06
Zuo et al., (2010) [35]	62%	0.007	Random	1.14 (0.97–1.33)	0.10
Tan et al., (2010) [33]	59%	0.01	Random	1.11 (0.94–1.30)	0.21
Lorenzo-Betancor et al., (2011) [34]	57%	0.02	Random	1.16 (0.99–1.35)	0.06
Wu et al., (2011) [37]	62%	0.008	Random	1.14 (0.98–1.33)	0.10
Bourasa et al., (2011) [39]	62%	0.007	Random	1.14 (0.97–1.34)	0.11
Radovica et al., (2011) [38]	62%	0.007	Random	1.13 (0.97–1.33)	0.11

ET, essential tremor; LINGO1, leucine-rich repeat and lg domain containing nogo receptor-interacting protein 1; OR, odds ratio; CI, confidence interval.

A few meta-analyses concerning the role of genetic variants at ET have been conducted [34,50,58,140,141,142,143]. Regarding *LINGO1* rs9652490 and *LINGO1* rs11856808, previous meta-analyses have reported association with ET [34,141,142]. The lack of the association in our meta-analysis it may be due the fact that we did not include data (neither from the discovery phase nor from the follow-up) from the GWAS from Stefansson et al. [19]. It also could be attributed to the high heterogeneity (I^2^ = 58%, P_Q_ = 0.01) that was observed in the current meta-analysis. For the *SLC1A2* rs3794087 our results are in accordance with previous meta-analyses [50,140,142], that failed to report any association, despite the fact that we did not used the GWAS from Their et al., [20] and we also include data from Ross et al. [54]. Finally, the marginal association between *STK32B* rs10937625 and ET should be interpreted with caution as the analysis based on only two studies in Asian populations.

## Conclusions

In the current review, we thoroughly reviewed 74 articles regarding genes and genetic loci that confer susceptibility to ET. Based on our results, over 50 genes/genetic loci have been examined for possible association with ET. Results from our meta-analyses suggest that LINGO1 rs9652490 and STK32B rs10937625 may influence, to some extent, ET susceptibility. However, despite the considerable number of studies that have been conducted and the significant effort made in order to identify genes of ET, consistently repeated results have yet to appear. These could be attributed, to some extent, to diagnostic difficulties (as the diagnosis is based on clinical evaluation) [144,145,146,147,148,149,150], heterogeneity among ancestry in studies, ethnicity, variability of the power of the sample sizes, different statistical and methodological approaches among studies, and other confounding factors.

Our study has some limitations. Firstly, we included studies without performing any quality assessment, in order to present the most accurate data possible. Moreover, the possibility that some eligible studies failed to be obtained through our search strategy is unlikely but cannot completely be excluded. Finally, the current review would have more robustness if more family, twin and whole exome studies regarding ET had included.

In view of the former considerations, collaborative studies with adjustment for other possible ET confounders (e.g. consumption of b-carboline alkaloid, caffeine and ethanol, harmane, exposure to pesticides, lead and other heavy metals, antioxidants, smoking and aging among others) are needed. In this way, the pathophysiological mechanisms of ET and the net effect of the genetic and environmental contribution to this entity could be revealed.

## Additional Files

The additional files for this article can be found as follows:

10.5334/tohm.67.s1 Supporting File 1. The complete search algorithm.

10.5334/tohm.67.s2 Supporting File 2. Baseline characteristics from the studies.

10.5334/tohm.67.s3 Supporting File 3. Forest plots from sensitivity analyses.
