# Supplementary material for: Genetic Risk Factors for Essential Tremor: A Review
Source: Tremor Other Hyperkinet Mov (N Y). 2020 Jun 11;10:4. doi: 10.5334/tohm.67 (PMC7394223; doi:10.5334/tohm.67)
Supplement: Supporting File 1. — The complete search algorithm. [file tohm-10-1-67-s1.pdf]

essential tremor polymorphism

("essential tremor"[MeSH Terms] OR ("essential"[All Fields] AND "tremor"[All Fields]) OR "essential tremor"[All Fields]) AND ("polymorphism, genetic"[MeSH Terms] OR ("polymorphism"[All Fields] AND "genetic"[All Fields]) OR "genetic polymorphism"[All Fields] OR "polymorphism"[All Fields])
