# Supplementary material for: Genetic Risk Factors for Essential Tremor: A Review
Source: Tremor Other Hyperkinet Mov (N Y). 2020 Jun 11;10:4. doi: 10.5334/tohm.67 (PMC7394223; doi:10.5334/tohm.67)
Supplement: Supporting File 2. — Baseline characteristics from the studies. [file tohm-10-1-67-s2.pdf]

Table 1: *Baseline characteristics of studies investigating the association gene risk factors and essential tremor. ("essential tremor" and "polymorphism")*											
					**Cases**			**Controls**			
**Author (Year)[Ref.]**	**Population**	**Tested Genes (Variants)**	**Including patients positive family history/HWE test/multiple test correction**	**Diagnosis Assessment**	**Mean age±SD/ Age of onset±SD**	**n**	**Male/ Female**	**Mean Age±SD**	**n**	**Male/ Female**	**Main Results & Comments**
Chen et al., (2019)[1]	Chinese	**HMOX1** (rs2071746), **HMOX2** (rs4786504, rs1051308), **VDR** (rs731236), **IL17A** (rs8193036), **IL1B** (rs1143643, rs1143634, rs1143633), **NOS1** (rs693534, rs7977109), **ADH1B** (rs6413413, rs1229984)	yes/yes(total cohort, cases and controls)/yes	Consensus Statement of the Movement Disorders Society on tremor in 1998 (Deuschl et al. 1998)	65.72±9.25/-	225	108/117	64.30 ±13.30	229	93/136	Rs1143633 of IL1B was associated with the risk of ET after adjusting for age and gender (recessive model), after multiple comparisons correction
Sazci et al., (2018)[2]	Caucasian	**VDR** (rs2228570)	-/yes (cases and controls)/no	Criteria of differential diagnosis assessment (Higgins et al. 1997, Shatunov et al. 2006)	57.2±19.9/45.2 ±12.8	239	129/110	58.4 ±15.80	239	129/110	The TT genotype of the rs2228570 variant was associated with SET (P = 0.033; OR = 0.453; 95%, CI = 0.216–0.952) and similarly, the C allele was associated with an increased risk SET (P =

											0.033; OR = 2.207; 95% CI= 1.051–4.636).
Xiao et al., (2018)[3]	Asian	**STK32B** (rs10937625), **PPARGC1A** (rs17590046)	yes/no/no	Consensus Statement of the Movement Disorders Society on tremor in 1998 (Deuschl et al. 1998)	51.20±20.0/-	469	264/205	53.1 ±9.00	470	256/214	Rs17590046 was associated with ET
Chen et al., (2018)[4]	Han Chinese	**LRRK1** (rs2924835) **LRRK2** (rs34594498, rs34410987, and rs33949390)	-/yes/no	Consensus Statement of the Movement Disorders Society on tremor in 1998 (Deuschl et al. 1998)	50.7±15.5/41.4 ±18.5	200	100/100	52.1± 16.5	434	217/217	Negative
Ng et al., (2018)[5]	Asian	**LRRK2** [rs7133914 (R1398H) and rs7308720 (N551K)]	-/no/no	Consensus Statement of the Movement Disorders Society on tremor in 1998 (Deuschl et al. 1998)	51.4±20.2/-	518	-	52.5±11.5	2,680	-	Negative
Agúndez et al., (2018)[6]	Caucasian Spanish	**ALAD** (rs1800435) for association analysis<br>**HMOX1** (rs2071746, rs2071747), **HMOX2** (rs2270363, rs1051308), for interaction analysis	yes/yes (cases and controls)/yes	Consensus Statement of the Movement Disorders Society on tremor in 1998 (Deuschl et al. 1998)	-/48.2±18.1	202	101/101	43.6 ±12.6	218	109/109	ALAD rs1800435 was not associated with the FET risk, but its interaction with the HMOX2 rs1051308 could be weakly associated with the FET

Emamalizadeh et al., (2017)[7]	Iranian	**RIT2** (rs12456492, rs16976358)	-/yes/no	Patients were diagnosed and confirmed by two neurologists	45.6±8.3/-	350	192/158	46.8±9.1	350	184/166	Rs12456492 was associated with ET
Zhang et al., (2017)[8]	Chinese	**STK32B** (rs10937625), **PPARGC1A** (rs17590046), **CTNNA3** (rs12764057, rs10822974 and rs7903491)	yes/yes/no	Diagnostic criteria for definite or probable ET (Louis et al. 1998)	36.23±15.93/46.73±17.04	218	113/105	36.87±10.46	315	168/147	C allele of rs10937625 was a protective factor and G allele of rs7903491 was a risk factor for ET.
Xu et al., (2016)[9]	Chinese	**SLC1A2** (rs3794087)	-/yes (controls)/yes	Diagnostic criteria for definite or probable ET (Louis et al. 1998)	49.98±19.20/39.05±18.83	112	59/53	55.99±11.60	437	220/217	Negative

Ortega-Cubero et al., (2015)[10]	Spain, Replication Cohort) Italy, Germany, North-America and Taiwan. Third step, an additional control group of 14777 individuals from European (Spanish, Italian, German).	**TREM2** (p.R47H-rs75932628)	yes/-/no	Consensus Statement of the Movement Disorders Society on tremor in 1998 (Deuschl et al. 1998)	68.43±13.82/51.44±19.15	1353	732/621	77.22±11.31	4168	1837/2331	Association in Spanish population. Did not replicate in other ethnicities.
Chao et al., (2015)[11]	90% Asian	**LRRK2** (R1628P)	yes/-/no	Consensus Statement of the Movement Disorders Society on tremor in 1998 (Deuschl et al. 1998)	Median: 55/40	450	251/199	Median: 54	827	422/405	Positive
Ayuso et al., (2015)[12]	Spanish white	**HMOX1** (rs2071746, rs2071747) **HMOX2** (rs2270363, rs1051308)	yes/yes (cases and controls)/yes	Consensus Statement of the Movement Disorders Society on tremor in 1998 (Deuschl et al. 1998)	65.7±16.1/48.2±18.1	202	100/102	63.6±14.6	747	379/368	Allelic frequencies of rs2071746T and rs1051308G were lower in ET patients.
Clark et al.,	Caucasian sample	**MAPT** (rs105255)	yes/yes/no	Louis ED, Jiang W, Pellegrino KM et al:	-	249	-	-	237	-	Negative

(2014)[13]	from North America			Elevated blood harmane (1-methyl-9H-pyrido[3,4-b]indole) concentrations in essential tremor. Neurotoxicology 2008; 29: 294–300							
Ross et al., (2014)[14]	North America (United States)	**SLC1A2** (rs3794087)	-/yes/no	Diagnostic criteria for definite or probable ET (Louis et al. 1998)	68.2±11.9/50.7 ±20.1	256	Ratio 1/1.1	65.3±12.8	726	Ratio 1/1.4	Negative
	North America (Canada)				73.3±13.7/55.3 ±17.7	179	Ratio 1/1.7	72.2±12.5	186	Ratio 1/2.2	Negative
Rajput et al., (2014)[15]	Canada	**FUS** (WES)	yes/-/-	Louis ED, Ford B, Lee H, Andrews H, Cameron G. Diagnostic criteria for essential tremor: a population perspective. Arch Neurol 1998; 55: 823–828	72.1±15.1/54.4 ±18.3	217	-	71.0±12.3	219	-	The non-pathogenic mutation p.G174_G175 del in one ET patient and two healthy controls, and a novel p.R377W in one patient with family history of disease were identified.

Zheng et al., (2013)[16]	Chinese	**FUS** [c.52C>A (p.P18T; rs144888138), c.147C>A (p.G49G; rs741810), c.291T>C (p.Y97Y; rs1052352), and c.684C>T (p.G228G; rs151073460)]	yes/yes/no	Consensus Statement of the Movement Disorders Society on tremor in 1998 (Deuschl et al. 1998)	41.3 ± 19.5/50.6±15.1	180	102/78	50.9±14.9	273	156/117	Negative
Ortega-Cubero et al., (2013)[17]	Spanish Controls: from 1000 Genomes project	**FUS/TLS** (rs61733962-c.153C>T, rs741810-c.147C>A, rs1052352-c.291C>T, rs147528034-c.636C>T, rs150529460-c.1464C>T, c.168T>G, rs72550890-c.670insGGC, rs929867-c.-54A>G, rs80301724-c.*41G>A, c.13+107insT, rs73530283-c.190+9T>C, rs72550862-c.833-29C>T, c.1293-37delC)	yes/no/no	NA	65.68±16.16/50.56±21.2	178	56.7% female	-	-	-	Negative
Wu et al., (2013)[18]	Chinese	**FUS** (Met392Ile)	yes/no/no	Consensus Statement of the Movement Disorders Society on tremor in 1998 (Deuschl et al. 1998)	50.6±20.1/37.8±21.2/	263	54.6% males	52.26±10.7	5,919	62.6% males	Met392Ile, in the FUS gene that increases susceptibility of ET among ethnic Chinese.
	Taiwan				60.5±14.9/55.5±17.1	250	46.4% males	61.1±13.6	250	48% males	
Labbe et al., (2013)[19]	non-Hispanic Caucasians of mixed European ancestry	**FUS** (p.R216C, p.Q290X and p.P431L)	-/no/no	Consensus Statement of the Movement Disorders Society on tremor in 1998 (Deuschl et al. 1998)	75±12/66±14	112	53/59	72±13	716	297/419	Negative
Sun et al.,	China	**GBA** (L444P)	yes/no/no	Consensus Statement of the	49.72±14.22/39.03±13.33	109	67/42	range 12–76 years	657	-	Negative

(2013)[20]				Movement Disorders Society on tremor in 1998 (Deuschl et al. 1998)							
Yu et al., (2013)[21]	Taiwanese	**SLC1A2** (rs3794087)	yes/no/no	Consensus Statement of the Movement Disorders Society on tremor in 1998 (Deuschl et al. 1998)	60.3±14.5/55.3 ±16.9	273	47.1% males	61.2±13.2	269	47.9% males	The SLC1A2 rs3794087 A allele was more frequent in ET patients compared to controls
García-Martín et al., (2013)[22]	Spanish	**SLC1A2** (rs3794087)	yes/yes/no	Consensus Statement of the Movement Disorders Society on tremor in 1998 (Deuschl et al. 1998)	65.7±16.1/48.2 ±18.1	202	100/102	46.5±12.6	308	151/157	Negative
Liang et al., (2013)[23]	Chinese Han	**LINGO1** (rs2271398, rs2271397, rs3743481, ss491228439)	-/yes/no	Consensus Statement of the Movement Disorders Society on tremor in 1998 (Deuschl et al. 1998)	51.0±18.2/41.7 ±19.1	151	87/64	52.6±17.8	301	176/125	Rs2271397, ss491228439 and the A465-C474-C714 haplotype were associated with increased ET risk in female.
García-Martín et al., (2013)[24]	Spanish	**DRD3** (312G > A-rs6280- Ser9Gly)	yes/yes/yes	Consensus Statement of the Movement Disorders Society on tremor in 1998 (Deuschl et al. 1998)	65.7±16.2/48.1 ±19.0	201	100/101	45.4±17.4	282	141/141	DRD3 genotype and the variant DRD3Gly allelic variant was associated with the risk

											for and age at onset of ET, and with the risk for voice tremor.
Tan et al., (2013)[25]	Chinese	**SLC1A2** (rs3794087)	yes/yes/no	Consensus Statement of the Movement Disorders Society on tremor in 1998 (Deuschl et al. 1998)	50.5±20.1/37.7	357	54%	58.8±9.6	1,93 5	51.1%	Positive
Parmalee et al., (2013)[26]	Non-Hispanic white	**FUS/TLS** (rs741810, rs1052352, rs2735393, rs4889537, Gly156Glu, Arg234Cys, Arg244Cys, Arg514Gly, rs121909667, rs121909669, rs121909671, Gly191Ser, Ser462Phe, rs139980267, rs141516414, rs17852338, rs12446646, rs150858484, rs61732970, rs144342946, rs41292386, rs3764326 , rs7194882, rs929867, rs79061794, rs72550895, rs186547381)	yes/yes/-	Louis ED, Ottman R, Ford B, et al. The Washington Heights-Inwood Genetic Study of Essential Tremor: methodologic issues in essential-tremor research. Neuroepidemiology 1997; 16: 124–133.	-/43.0±23.1	259	136/123	-	262	113/149	Negative
García-Martín et al., (2012)[27]	Spanish	rs1052553, which discriminates between **MAPT H1 and H2 haplotypes**	yes/yes/no	Consensus Statement of the Movement Disorders Society on tremor in 1998 (Deuschl et al. 1998)	65.71±16.1/48.2±18.1	200	99/101	46.5±12.6	291	146/145	Negative
Merner et al., (2012)[28]		**FUS** (p.Arg216Cys, p.Pro431Leu)	yes/-/-	Louis, E.D., Ford, B., Lee, H., Andrews, H., and Cameron, G. (1998). Diagnostic criteria for	-	270	-	-	450	-	p.Arg216Cys, variant was detected in two ET cases, one with

				essential tremor: a population perspective. Arch Neurol 55, 823-828. Deuschl, G., Bain, P., and Brin, M. (1998). Consensus statement of the Movement Disorder Society on Tremor. Ad Hoc Scientific Committee. Mov Disord 13 Suppl 3, 2-23							familial ET and the other a sporadic case. p.Pro431Leu in a case with familial ET
Liang et al., (2012)[29]	Chinese Han	**LINGO4** (rs61746299 and rs1521179)	yes/yes/yes	Consensus Statement of the Movement Disorders Society on tremor in 1998 (Deuschl et al. 1998)	51.8±18.1/41.6 ±19.3	150	85/65	52.3±16.6	300	173/127	Negative
Radovica et al., (2012)[30]	Latvian	**LINGO 1** (rs2137110, rs8030859, rs74844064, rs34476171, rs9652490, rs7177008, rs13313467, rs8028808, rs11856808 and rs72744599)	yes/yes/no	By a neurologist with a specific expertise in movement disorders	-	218	-	-	130	-	The A/G genotype of the rs96532490 was associated with FET compared with controls.
Thier et al., (2011)[31]	German and Danish	**15 GABAAR and 4 GABA transporter genes**	yes/yes/no	Consensus Statement of the Movement Disorders Society on tremor in 1998 (Deuschl et al. 1998)	184: 70.74±11.22/63 .59±10.18<br>319: 60.35±15.90/31 .11±20.51	503 (184 +319)	103/81<br>190/129	426: 65.61±7.51<br>392: 70.21±5.92	818 (426 +39 2)	146/280<br>211/181	Negative
Villarino -Guell et al., (2011)[32]	North American Caucasian s	**MAPT H1 discriminating SNP** (rs1052553), **H1c subhaplotype SNP (**rs242557)	-/no/no	NA	71.3±12.4/52.7 ±19.2	356	151/205	72.2±10.8	409	215/194	MAPT H1 was associated with ET

García-Martín et al., (2011)[33]	Controls: Caucasian Spanish	**GABRR1** (M26V-Met26Val, rs12200969), **GABRR1** (H27R-His26Arg, rs1186902), **GABRR2** (T455M-Thr55Met, rs282129), and **GABRR3** (Y205X-Tyr205X, rs832032)	yes/yes/yes	Consensus Statement of the Movement Disorders Society on tremor in 1998 (Deuschl et al. 1998)	65.7±16.1/48.2 ±18.1	200	99/101	45.5±12.2	250	125/125	Negative
García-Martín et al., (2011)[34]	Controls: Caucasian Spanish	**GABRA4** (L26M -Leu26Met, rs2229940), **GABRE** (S102A-Ser26Ala, rs1139916) and **GABRQ** (I478F-Ile26Phe, rs3810651)	yes/yes/yes	Consensus Statement of the Movement Disorders Society on tremor in 1998 (Deuschl et al. 1998)	65.7±16.1/48.2 ±18.1	200	99/101	45.5±12.2	250	125/125	Negative
Wu et al., (2011)[35]	Asian (Singapore)	**LINGO2** (rs10812774, rs10968280, rs1412229, and rs7033345, rs17506843, rs111772526, rs112609154, rs113646467)	-/yes in controls/yes	Consensus Statement of the Movement Disorders Society on tremor in 1998 (Deuschl et al. 1998)	52.0±18.6/41.4 ±20.2	327	174/153	65.7±9.3	499	258/241	The rs7033345 and rs10812774 were associated with ET
Bourasa et al., (2011)[36]	French-Canadian	**LINGO1** (rs9652490 and rs11856808)	yes/yes/yes	NA	-	259	-	-	479	-	Negative
Wu et al., (2011)[37]	Asian	**LINGO1** (rs9652490)	yes/yes in controls/no	Consensus Statement of the Movement Disorders Society on tremor in 1998 (Deuschl et al. 1998)	56.52±14.73/45.99±17.98	117	67/50	59.25±10.28	160	84/76	Negative
Lorenzo-Betancor et al., (2011)[38]	Spain	**LINGO1** (rs9652490 and rs11856808)	yes/yes/no	Consensus Statement of the Movement Disorders Society on tremor in 1998 (Deuschl et al. 1998)	65.69±15.59/49.12±18.96	226	49.1% female	63.59±14.55	1,117	49.9% female	Negative
Ross et	North	**SNCA** (rs356218, rs17180453,	-/yes/no	Consensus Statement	Total	661			131		Negative

al., (2011)[39]	American (Caucasian and African-Amenrican)	rs3775423, rs2736990, rs2572324, rs3796661, rs2737033, rs3775439, rs10014396, rs9995651, rs2583959, rs2737012, rs1372519, rs3756063, rs1372520, rs2619361, rs2619362, rs2301135, rs2619363, rs2583988)		of the Movement Disorders Society on tremor in 1998 (Deuschl et al. 1998)<br><br>Louis ED, Ford B, Lee H, Andrews H, Cameron G. Diagnostic criteria for essential tremor: a population perspective. Arch Neurol 1998;55:823–828					6		
					Mayo: 72.8±10.7/50.1 ±20.2	135	66/69	72.1±10.9	427	228/199	
					Canada: 70.0±13.3/54.3 ±18.5	201	75/126	67.8±13.2	313	90/223	
					Emory: 69.6±12.3/46.0 ±21.7	118	51/67	76.0±6.9	268	102/166	
					Columbia: 69.9±13.7/44.5 ±23.2	193	90/103	67.5±11.8	282	118/164	
Zahorakova et al., (2010)[40]	Czech	**ETM2 locus** [3 STRs (etm1231, etm1234, and etm1240)]	yes/no/yes	Jankovic J (2000). Essential tremor: clinical characteristics. Neurology. 54: S21–25	65±15/-	61	27/34	67±9	68	29/39	Negative
Zuo et al., (2010)[41]	Chinese	**LINGO1** (rs9652490 and rs11856808)	yes/yes/no	Consensus Statement of the Movement Disorders Society on tremor in 1998 (Deuschl et al. 1998)	49.7±14.2/39.0 ±13.3	109	-	49.5±15.8	430	-	Negative
Clark et al., (2010)[42]	North Americans (Non-Hispanic whites)	**LINGO1** (rs3144, rs11853396, rs3743481, rs2271396, rs2271397, rs2271398, rs11633842, rs9652490, rs11631120, rs7176315, rs7177008, rs13313467, rs8028808, rs11856808, rs11856876)	yes/yes in controls/yes	Louis ED, Jiang W, Pellegrino KM et al: Elevated blood harmane (1-methyl-9H-pyrido[3,4-b]indole) concentrations in essential tremor. Neurotoxicology 2008; 29: 294–300	-/43.8±22.7	257	134/122	-	265	113/152	Rs9652490 was associated with ‘definite’ or ‘probable’ ET (p=0.03, OR=1.41). Rs177008, rs13313467 and rs8028808, were associated

											with early-onset ET (p=0.028, OR=1.52; p=0.0238, OR=1.54; and P=0.0391, OR=1.55, respectively).
Villarino -Guell et al., (2010)[43]	Caucasian origin from North America	**LINGO1** (rs4886887, rs3144, rs3743481, rs907396, rs907400, rs11856978, rs7162113, rs13329256, rs9652490, rs8028808, rs11855874, rs4886893, rs4886894, rs12898861, rs4243047, rs12905478) **LINGO2** (rs10968215, rs9644872, rs13362909, rs10757699, rs7854367, rs4880001, rs10968280, rs11793421, rs10812774, rs16912763, rs1331866, rs13296489, rs10968542, rs16912778, rs2026376, rs10757744, rs1412229, rs4879257, rs7033345, rs1438478, rs6476092)	yes/yes/no	Louis ED, Ford B, Lee H, Andrews H, Cameron G (1998) Diagnostic criteria for essential tremor: a population perspective. Arch Neurol 55:823–828	67±15/50±20	1,247	530/717	73±10	642	310/332	Rs9652490 rs4886887, rs3144, rs8028808, and rs12905478 (LINGO1) and rs1412229 (LINGO2) were associated with ET. Rs907396 (LINGO1) and rs10812774, rs7033345 (LINGO2) were influenced age at onset of ET.
Thier et al., (2010)[44]	European (German and French)	**LINGO1** (rs907389, rs9806139, rs2271396, rs8024724, rs2292417, rs8030859, rs9652490, rs11856808 rs7178761, rs4243047)	yes/yes/yes	Consensus Statement of the Movement Disorders Society on tremor in 1998 (Deuschl et al. 1998)	German: 62±15/33±21	284	180/104	69.2±5.5	334		Rs8030859, rs9652490, and rs11856808 were associated with ET in
					French: 55±19/29±20	48	23/25		240		

											Germans, and rs9652490 and rs11856808 were associated with ET in French.
Tan et al., (2010)[45]	Asian	**LINGO1** (rs9652490)	yes/yes in controls/no	Consensus Statement of the Movement Disorders Society on tremor in 1998 (Deuschl et al. 1998)	Median: 52.6/41.0	190	-	Median: 56.7	733	-	G allele of the rs9652490 was associated familial ET. An individual with two G alleles has more than 3 times risk of having ET.
Clark et al., (2010)[46]	Non-Hispanic whites	**LRRK2** (G2019S, I2020T, R1441C, Y1699C, L1114L, I1122V and 19 LRRK2 SNPs)	yes/yes/yes	Louis ED, Jiang W, Pellegrino KM, Rios E, Factor-Litvak P, Henchcliffe C, et al. Elevated blood harmane (1-methyl-9H-pyrido[3,4-b]indole) concentrations in essential tremor. Neurotoxicology 2008;29(2):294–300	67.3±15.0/-	275	142/133	67.7±11.1)	289	121/168	Negative
	Ashkenazi Jewish	**GBA** (all exons sequenced)	yes/yes/yes	Additional Brain autopsy for 24 ET brains	68.4±15.8/-	93/and 24 ET brains	41/52	70.2±11.9	62	26/38	Negative
García-Martín et al., (2010)[47]	Controls: Caucasians Spanish	**PON1** (Leu55Met and Glu192Arg)	yes/yes/yes	Consensus Statement of the Movement Disorders Society on tremor in	65.7±16.1/48.2 ±18.1	201	100/100	45.4±12.2	220	110/100	Negative

				1998 (Deuschl et al. 1998)								
Keeling et al., (2010)[48]	North American Caucasian	**HNMT** (Thr105Ile-rs11558538)	-/yes/no	Louis ED, Ford B, Lee H, Andrews H, Cameron G. Diagnostic criteria for essential tremor: a population perspective. Arch Neurol 1998;55(6): 823–8	70.9/52.5±19.3	338	1/0.8 female to male	72.2	409	1/1.1 female to male	Negative
Villarino-Guell et al., (2010)[49]	North America Caucasians	**LINGO1** (rs9652490)	yes/yes /no	Louis ED, Ford B, Lee H, Andrews H, Cameron G. Diagnostic criteria for essential tremor: a population perspective. Arch Neurol 1998;55(6):823–8.	70.9±13.3/52.8±19.1	356	US:1/0.97 male to female<br>Canada: 1/1.67 male to female	72.2±10.8	428	1/0.91 male to female	Positive
Lorenz et al., (2009)[50]	Germany, Denmark, France	**DRD3** (312G > A-rs6280- Ser9Gly)	yes/yes/yes	Consensus Statement of the Movement Disorders Society on tremor in 1998 (Deuschl et al. 1998)	58.7±16.1/29.4±19.2<br>62.4±15.7/30.6±21.1	202 familial cases<br>97 sporadic cases	119/83<br>57/40	-	528	299/229	Negative
Inashkina et al., (2008)[51]	Latvian, Russian, Byelorussians, Ukrainians, Polish, Lithuanians	**ETM1 ETM2 loci** (STRs)<br>**DRD3** (312G > A-rs6280- Ser9Gly)	yes/-/no	By a neurologist with a specific expertise in movement disorders.	62.5±15.8/-	104	23%/73%	-	116	-	The highest difference of frequencies was found in allele 171 of the marker D2S220 (OR 0.13, 95% CI 0.02–1.03, P = 0.05). Negative for the rs6280.

Ledesma et al., (2008)[52]	Caucasian Spanish	**HNMT** (Thr105Ile- rs11558538)	-/yes /yes	Consensus Statement of the Movement Disorders Society on tremor in 1998 (Deuschl et al. 1998)	48.1±18.9/-	204	102/102	37.5±14.6	295	135/160	Patients with ET showed a higher frequency of homozygous HNMT 105Thr genotypes.
Tan et al., (2008)[53]	Asian	**LRRK2** (G2385A)	yes/-/no	Consensus Statement of the Movement Disorders Society on tremor in 1998 (Deuschl et al. 1998)	52.1±19.6/41.8 ± 21.6	172	53.0%male	62.2 ±11.6	247	54.3% male	Negative
Vitale et al., (2008)[54]	Italian	**DRD3** (312G > A-rs6280- Ser9Gly)	yes/yes /yes	Consensus Statement of the Movement Disorders Society on tremor in 1998 (Deuschl et al. 1998)	64.7±15.3/50.32±18.5	116	60/56	63.5±16.7	158	81/77	Negative
Chen et al., (2008)[55]	Han Chinese in Taiwan	CAG repeats in the **PPP2R2B gene**	-/yes in controls/yes	Findley LJ, Koller WC. 1995. Definitions and behavioral classifications. In: Findley LJ, Koller WC, editors. Handbook of tremor disorders. New York: Marcel Dekker. pp. 1–5.	63.4±14.4/-	132	48.6% females	57.2±15.1	625	47% females	The proportion of subjects carrying rare short $(CAG)_{5–7}$ alleles was higher in ET patients (4/132 [3.0%], p<0.001) as compared the control subjects (1/625 [0.2%]).

Blair et al., (2008)[56]	Caucasians	**DRD3** (312G > A-rs6280- Ser9Gly)	yes/yes /no	Jankovic J. Essential tremor: clinical characteristics. Neurology 2000;54(Suppl):S21e5.<br><br>Jankovic J. Essential tremor: a heterogeneous disorder. Mov Disord 2002;17:638e44	47±19.77/43±17.69	237	100/137	Unrelated controls:66.33±5.34	121	58/63	Negative
								Family controls:54±12.67	151	65/86	
Martinez et al., (2008)[57]	Caucasian Spanish	**GSTP1** (Ile105Val -rs1695)	yes/yes /no	Consensus Statement of the Movement Disorders Society on tremor in 1998 (Deuschl et al. 1998)	65.7±16.1/48.2±18.1	200	100/100	45.5±12.2	220	110/110	Mutated allelic variants were significantly more frequent in individuals with ET exposed to pesticides compared with non-exposed patients.
Martinez et al., (2007)[58]	Spanish	**ADH2** (A213G-rs1229984)	yes/yes /no	Consensus Statement of the Movement Disorders Society on tremor in 1998 (Deuschl et al. 1998)	65.7±16.2/48.1±19.0	204	102/102	45.4±17.4	200	100/100	Negative
Martinez et al., (2007)[59]	Caucasian Spanish	**CYP2C** (CYP2C8 and CYP2C9 allelic variants)	yes/yes /no	Koller W. C. and Busenbark K. L. (1997) Essential tremor. In: Watts R. L., Koller W. C., eds. Movement Disorders.	65.7±16.2/48.1±19	200	100/100	44.6±14.4	300	150/150	Alterations at the CYP2C gene locus are associated with the risk

				New York, McGraw-Hill, 365−385							for ET.
Tan et al., (2007)[60]	Asian	**DRD3** (Ser9Gly)	yes/yes /no	movement disorder neurologist	56.0/43.5	163	86/77	62.0	192	85/107	Negative
Alonso-Navaro et al., (2006)[61]	Caucasians	**CYP2C19**	yes/yes /no	Koller WC, Busenbark KL: Essential tremor; in Watts RL, Koller WC (eds): Movement Disorders. New York, McGraw-Hill, 1997, pp 365–385.	65.7±16.2/48.1 ±19.0	200	100/100	44.6±14.4	300	150/150	Heterozygosis CYP2C19*1/CYP2C19*2 is associated with the risk for ET.
Deng et al., (2006)[62]	North America	**GABRA1** [156T > C (nt 6090903, NT 023133)]	yes/yes /no	L.J. Findley, W.C. Koller, Definitions and behavior classifications, in: L.J. Findley, W.C. Koller (Eds.), Handbook of Tremor Disorders, Marcel Dekker, New York, 1994, pp. 1–5	53.1±18.9/35.8 ±19.8	121	57/64	53.4±16.3	114	47/67	Negative
Lucotte et al., (2006)[63]	French	**DRD3** (312G > A-rs6280- Ser9Gly)	yes/-/-	Consensus Statement of the Movement Disorders Society on tremor in 1998 (Deuschl et al. 1998)	-	30	-	-	50	-	Positive

Jeanneteau et al., (2006)[64]	Caucasians	**DRD3** (312G > A-rs6280- Ser9Gly)	-/yes (controls)/no	Consensus Statement of the Movement Disorders Society on tremor in 1998 (Deuschl et al. 1998)	Range: 14-93/3-83	276	126/150	Range: 9-88	184	83/101	Association with risk and age-at-onset of ET.
Deng et al., (2005)[65]	North America	**HS1BP3** (A265G)	yes/yes /no	Findley LJ, Koller WC. Definitions and behavior classifications. In: Findley LJ, Koller WC, eds. Handbook of tremor disorders. New York: Marcel Dekker, 1994:1–5.	58.1±14.6/-	222	114/108	60.1±14.4	132	68/64	Negative
Louis et al., (2005)[66]	non-Hispanic white, non-Hispanic African American, Hispanic	**ALAD**	yes/-/no	Louis ED, Ford B, Frucht S, Barnes LF, Tang M-X, Ottman R. Risk of tremor and impairment from tremor in relatives of patients with essential tremor: a community-based family study. Ann Neurol 2001;49:761–769	67.4±16.6/-	63	42.9% female	64.9±11.1	101	56.4% female	Negative. ET case were higher in individuals with both an ALAD-2 allele and an elevated BPb concentration than in individuals with only an elevated BPb concentration
Sazci et al., (2004)[67]	Caucasian	**MTHFR** (C677T and A1298C)	-/yes/no	Bain P, Brin M, Deuschl G, et al. Criteria for the diagnosis of essential tremor. Neurology 2000;54(Suppl. 4):S7. Deuschl G. Differential diagnosis of tremor. J	54.41±18.48/34.48±22.39	158	77/81	53.35±15.94	246	120/126	677T, 1298C alleles and T677T genotype and T677T/A1298A, and C677C/C1298C compound

				Neural Transm Suppl 1999;56:211–220							genotypes were associated with ET.
Pigullo et al., (2003)[68]	Italian	**SNCA/a-synuclein** (NACP-Rep1, rs1372520, rs1372519, rs1372518, SNP273, SNP15)	yes/yes/no	Findley LJ, Koller WC. Definition and behavioral classification. In: Findley LJ, Koller WC, editors. Handbook of tremor disorders. New York, NY: Marcel Dekker, Inc; 1995. p 1–6	65.8±14.4/49.5 ±18.4	106	-	-	90	-	Negative
Tan et al., (2000)[69]	white	**SNCA/a-synuclein** (NACP-Rep1-alleles 257bp 259bp 261bp 263bp)	-/-/yes	Consensus Statement of the Movement Disorders Society on tremor in 1998 (Deuschl et al. 1998)	63.2±14.0/	46	-	57.1±12.2	100	-	Allele 263bp was more frequent in ET patients
Pigullo et al., (2000)[70]	Italians	GAG expansions **hSKCa3** and **CACNL1A4**	yes/-/no	Findley LJ, Koller WC (1994) Definition and behavioral classification. In: Handbook of tremor disorders. Dekker, New York, pp 1–5	63.2±17.8/43.6 ±21.5	hSKCa3: 88<br>CACNLA 1A4: 98	-	-	hSK Ca3: 78<br>CAC NLA 1A4: 92	-	Negative
Agunez et al., (1997)[71]	White Spanish	**CYP2D6** (8 variants)	yes/yes/-	NA	61.3±12.9/47±1 8	91	39/52	45.4±12.9	258	117/141	Negative
Stefansson et	Discovery: Iceland	**GWAS**	yes/yes/-	Bain, P.G. et al. A study of hereditary essential	52(median)/20(median)	453	47%male	55(median)	14,394	44%male	rs9652490 and the

al., (2009)[72]	Follow-up:			tremor. Brain 117 (Pt 4), 805-24 (1994)							rs11856808, on LINGO1 gene, were associated with ET ((in discovery, all follow-up and all-combined samples). For the rs11856808 the significance was lost for the follow-up sample, after adjusting for the effect of rs9652490.
	Austria			Consensus Statement of the Movement Disorders Society on tremor in 1998 (Deuschl et al. 1998)	66.5(median)/40(median)	77	54%male	41(median)	432	60%male	
	Germany			by movement disorder specialists	60(median)/50(median)	69	45%male	48(median)	176	48%male	
	US<br>Iceland			Consensus Statement of the Movement Disorders Society on tremor in 1998 (Deuschl et al. 1998) and TRG criteria	70(median)/52(median)	122	43%male	66(median)	614	52%male	
Thier et al., (2012)[73]	Stage1-German-(Kiel) Stage2	**GWAS**	yes/yes/yes	Consensus Statement of the Movement Disorders Society on tremor in 1998 (Deuschl et al. 1998) and TRG criteria	60±16/37±22	436	277/159	53±15	928	494/434	The rs3794087 was associated with ET. in both stages of the GWAS, as well in the subgroup analysis in definite only ET patients
	German-(Kiel)				65±14/42±22	411	237/174	67±5	553	152/401	
	Danish				75±5/-	29	17/12	78±4	56	27/29	
	German-(TÜ)				69±17/51±19	50	21/29	-	-	-	
	Austrian				68±12/45±21	64	37/27	-	-	-	
Muller et al., (2016)[74]	Discovery stage: Europe	**GWAS**	yes/yes/yes	Consensus Statement of the Movement Disorders Society on tremor in 1998 (Deuschl et al.	62.34±14.64/39.41±21.95	887	37.1% female	50.37±14.13	4,063	49.8% female	The rs10937625 (STK32B) and the rs17590046 (PPARGC1A)
	North America				69.88±14.45/50.20±22.73	891	53.2% female	NA	773	56.0% female	

				1998), TRG criteria, Movement Disorder Specialist, Senior Neurologist							were associated with ET and the rs12764057, rs10822974 and rs7903491 (CTNNA3) were significant in the combined analysis of both stages.
	Replication Stage: Europe				63.53±15.19/42.00±22.31	425	49.6% female	42.24±17.22	431	44.3% female	
	North America				69.06±14.70/49.63±21.16	604	54.4% female	71.49±12.53	634	67.7% female	

ET, essential tremor; RLS, restless legs syndrome; SET, sporadic essential tremor; FET, familial essential tremor; HWE, Hardy-Weinberg equilibrium; SNP, single nucleotide polymorphism; CI, confidence interval; OR, odds ratio; NA, non-available; HMOX1, Heme Oxygenase 1; HMOX2, Heme Oxygenase 2; VDR , vitamin D receptor; IL17A, Interleukin-17A; IL1B, Interleukin-1B; NOS1, nitric oxide synthase 1; ADH1B, Alcohol dehydrogenase 1B; PPARGC1A, Peroxisome Proliferator-Activated Receptor Gamma Coactivator 1-Alpha; RIT2, Ras like without CAAX 2; ALAD, aminolevulinate dehydratase; STK32B, serine/threonine kinase 32B; CTNNA3, catenin alpha 3; SLC1A2, solute carrier family 1 member 2;MAPT, microtubule associated protein tau; LINGO1, leucine-rich repeat and lg domain containing nogo receptor-interacting protein 1; GABRR1, Gamma-Aminobutyric Acid Type A Receptor Rho1 Subunit; GABRR2, Gamma-Aminobutyric Acid Type A Receptor Rho2 Subunit; GABRR3, Gamma-Aminobutyric Acid Type A Receptor Rho3 Subunit; ADH2, Alcohol Dehydrogenase 2; LINGO4, Leucine-Rich Repeat And Immunoglobulin-Like Domain-Containing Nogo Receptor-Interacting Protein; FUS/TLS, Fused in Sarcoma/Translocated in Liposarcoma; GABAAR, gamma-aminobutyric acid type A receptors; GABA, gamma-aminobutyric acid; LINGO2, Leucine-Rich Repeat And Immunoglobulin-Like Domain-Containing Nogo Receptor-Interacting Protein 2; (STRs), short tandem repeats; PON1, Paraoxonase 1; HNMT, Histamine N-Methyltransferase; DRD3, dopamine D3 receptor; PPP2R2B, protein phosphatase 2 regulatory subunit Bbeta; GSTP1, glutathione S-transferase pi 1; CYP2C19 gene, cytochrome P450 family 2 subfamily C member 19; HS1BP3, HCLS1 binding protein 3; MTHFR, methylenetetrahydrofolate reductase; TREM2, triggering receptor expressed on myeloid cells 2; LRRK1, leucine-rich repeat kinase-1; LRRK2, leucine-rich repeat kinase-2; GBA, Glucocerebrosidase; WES, whole exome sequencing; SNCA, a-synuclein; CYP2D6, cytochrome P450 family 2 subfamily D member 6; hSKCa3, human small conductance calcium-activated potassium channel; CACNL1A4, calcium voltage-gated channel subunit alpha1 A; GWAS, genome-wide association study; TÜ, Tübingen.

References

1. Chen J, Huang P, He Y, Shen J, Du J, Cui S, et al. IL1B polymorphism is associated with essential tremor in Chinese population. BMC neurology. 2019;19(1):99.
2. Sazci A, Uren N, Idrisoglu HA, Ergul E. The rs2228570 Variant of the Vitamin D Receptor Gene is Associated with Essential Tremor. Neuroscience bulletin. 2019;35(2):362-4.
3. Xiao B, Deng X, Ng EY, Tio M, Prakash KM, Au WL, et al. GWAS-linked PPARGC1A variant in Asian patients with essential tremor. Brain : a journal of neurology. 2017;140(4):e24.
4. Chen H, Yuan L, Song Z, Deng X, Yang Z, Gong L, et al. Genetic Analysis of LRRK1 and LRRK2 Variants in Essential Tremor Patients. Genetic testing and molecular biomarkers. 2018;22(6):398-402.
5. Ng ASL, Ng EYL, Tan YJ, Prakash KM, Au WL, Tan LCS, et al. Case-control analysis of LRRK2 protective variants in Essential Tremor. Scientific reports. 2018;8(1):5346.
6. Agundez JAG, Garcia-Martin E, Alonso-Navarro H, Ayuso P, Esguevillas G, Benito-Leon J, et al. Delta-amino-levulinic acid dehydratase gene and essential tremor. European journal of clinical investigation. 2017;47(5):348-56.
7. Emamalizadeh B, Jamshidi J, Movafagh A, Ohadi M, Khaniani MS, Kazeminasab S, et al. RIT2 Polymorphisms: Is There a Differential Association? Molecular neurobiology. 2017;54(3):2234-40.
8. Zhang Y, Zhao Y, Zhou X, Li K, Yi M, Guo J, et al. Assessment of Three New Loci from Genome-wide Association Study in Essential Tremor in Chinese population. Scientific reports. 2017;7(1):7981.
9. Xu Y, Cao B, Chen Y, Ou R, Wei Q, Yang J, et al. SLC1A2 rs3794087 are associated with susceptibility to Parkinson's disease, but not essential tremor, amyotrophic lateral sclerosis or multiple system atrophy in a Chinese population. Journal of the neurological sciences. 2016;365:96-100.
10. Ortega-Cubero S, Lorenzo-Betancor O, Lorenzo E, Agundez JA, Jimenez-Jimenez FJ, Ross OA, et al. TREM2 R47H variant and risk of essential tremor: a cross-sectional international multicenter study. Parkinsonism & related disorders. 2015;21(3):306-9.
11. Chao YX, Ng EY, Tan L, Prakash KM, Au WL, Zhao Y, et al. Lrrk2 R1628P variant is a risk factor for essential tremor. Scientific reports. 2015;5:9029.
12. Ayuso P, Agundez JA, Alonso-Navarro H, Martinez C, Benito-Leon J, Ortega-Cubero S, et al. Heme Oxygenase 1 and 2 Common Genetic Variants and Risk for Essential Tremor. Medicine. 2015;94(24):e968.
13. Clark LN, Liu X, Parmalee NL, Hernandez N, Louis ED. The microtubule associated protein tau H1 haplotype and risk of essential tremor. European journal of neurology. 2014;21(7):1044-8.

14. Ross JP, Rayaprolu S, Bernales CQ, Soto-Ortolaza AI, van Gerpen J, Uitti RJ, et al. SLC1A2 rs3794087 does not associate with essential tremor. Neurobiology of aging. 2014;35(4):935.e9-10.
15. Rajput A, Rajput AH, Rajput ML, Encarnacion M, Bernales CQ, Ross JP, et al. Identification of FUS p.R377W in essential tremor. European journal of neurology. 2014;21(2):361-3.
16. Zheng W, Deng X, Liang H, Song Z, Gao K, Yang Y, et al. Genetic analysis of the fused in sarcoma gene in Chinese Han patients with essential tremor. Neurobiology of aging. 2013;34(8):2078.e3-4.
17. Ortega-Cubero S, Lorenzo-Betancor O, Lorenzo E, Alonso E, Coria F, Pastor MA, et al. Fused in Sarcoma (FUS) gene mutations are not a frequent cause of essential tremor in Europeans. Neurobiology of aging. 2013;34(10):2441.e9-.e11.
18. Wu YR, Foo JN, Tan LC, Chen CM, Prakash KM, Chen YC, et al. Identification of a novel risk variant in the FUS gene in essential tremor. Neurology. 2013;81(6):541-4.
19. Labbe C, Soto-Ortolaza AI, Rayaprolu S, Harriott AM, Strongosky AJ, Uitti RJ, et al. Investigating the role of FUS exonic variants in essential tremor. Parkinsonism & related disorders. 2013;19(8):755-7.
20. Sun QY, Guo JF, Han WW, Zuo X, Wang L, Yao LY, et al. Genetic association study of glucocerebrosidase gene L444P mutation in essential tremor and multiple system atrophy in mainland China. Journal of clinical neuroscience : official journal of the Neurosurgical Society of Australasia. 2013;20(2):217-9.
21. Yu SW, Chen CM, Chen YC, Chang CW, Chang HS, Lyu RK, et al. SLC1A2 variant is associated with essential tremor in Taiwanese population. PloS one. 2013;8(8):e71919.
22. Garcia-Martin E, Martinez C, Alonso-Navarro H, Benito-Leon J, Lorenzo-Betancor O, Pastor P, et al. No association of the SLC1A2 rs3794087 allele with risk for essential tremor in the Spanish population. Pharmacogenetics and genomics. 2013;23(11):587-90.
23. Liang H, Song Z, Deng X, Xu H, Zhu A, Zheng W, et al. Genetic analysis of the leucine-rich repeat and lg domain containing Nogo receptor-interacting protein 1 gene in essential tremor. Journal of molecular neuroscience : MN. 2013;51(2):403-7.
24. Garcia-Martin E, Martinez C, Alonso-Navarro H, Benito-Leon J, Puertas I, Rubio L, et al. Dopamine receptor D3 (DRD3) genotype and allelic variants and risk for essential tremor. Movement disorders : official journal of the Movement Disorder Society. 2009;24(13):1910-5.
25. Tan EK, Foo JN, Tan L, Au WL, Prakash KM, Ng E, et al. SLC1A2 variant associated with essential tremor but not Parkinson disease in Chinese subjects. Neurology. 2013;80(17):1618-9.
26. Parmalee N, Mirzozoda K, Kisselev S, Merner N, Dion P, Rouleau G, et al. Genetic analysis of the FUS/TLS gene in essential tremor. European journal of neurology. 2013;20(3):534-9.
27. Garcia-Martin E, Martinez C, Alonso-Navarro H, Benito-Leon J, Lorenzo-Betancor O, Pastor P, et al. H1-MAPT and the risk for familial essential tremor. PloS one. 2012;7(7):e41581.

28. Merner ND, Girard SL, Catoire H, Bourassa CV, Belzil VV, Riviere JB, et al. Exome sequencing identifies FUS mutations as a cause of essential tremor. American journal of human genetics. 2012;91(2):313-9.
29. Liang H, Zheng W, Xu H, Lei J, Song Z, Jiang X, et al. No evidence of association between the LINGO4 gene and essential tremor in Chinese Han patients. Parkinsonism & related disorders. 2012;18(3):303-5.
30. Radovica I, Inashkina I, Smeltere L, Vitols E, Jankevics E. Screening of 10 SNPs of LINGO1 gene in patients with essential tremor in the Latvian population. Parkinsonism & related disorders. 2012;18(1):93-5.
31. Thier S, Kuhlenbaumer G, Lorenz D, Nothnagel M, Nebel A, Christensen K, et al. GABA(A) receptor- and GABA transporter polymorphisms and risk for essential tremor. European journal of neurology. 2011;18(8):1098-100.
32. Vilarino-Guell C, Soto-Ortolaza AI, Rajput A, Mash DC, Papapetropoulos S, Pahwa R, et al. MAPT H1 haplotype is a risk factor for essential tremor and multiple system atrophy. Neurology. 2011;76(7):670-2.
33. Garcia-Martin E, Martinez C, Alonso-Navarro H, Benito-Leon J, Lorenzo-Betancor O, Pastor P, et al. Gamma-aminobutyric acid (GABA) receptor rho (GABRR) polymorphisms and risk for essential tremor. Journal of neurology. 2011;258(2):203-11.
34. Garcia-Martin E, Martinez C, Alonso-Navarro H, Benito-Leon J, Lorenzo-Betancor O, Pastor P, et al. Gamma-aminobutyric acid GABRA4, GABRE, and GABRQ receptor polymorphisms and risk for essential tremor. Pharmacogenetics and genomics. 2011;21(7):436-9.
35. Wu YW, Prakash KM, Rong TY, Li HH, Xiao Q, Tan LC, et al. Lingo2 variants associated with essential tremor and Parkinson's disease. Human genetics. 2011;129(6):611-5.
36. Bourassa CV, Riviere JB, Dion PA, Bernard G, Diab S, Panisset M, et al. LINGO1 variants in the French-Canadian population. PloS one. 2011;6(1):e16254.
37. Wu YW, Rong TY, Li HH, Xiao Q, Fei QZ, Tan EK, et al. Analysis of Lingo1 variant in sporadic and familial essential tremor among Asians. Acta neurologica Scandinavica. 2011;124(4):264-8.
38. Lorenzo-Betancor O, Garcia-Martin E, Cervantes S, Agundez JA, Jimenez-Jimenez FJ, Alonso-Navarro H, et al. Lack of association of LINGO1 rs9652490 and rs11856808 SNPs with familial essential tremor. European journal of neurology. 2011;18(8):1085-9.
39. Ross OA, Conneely KN, Wang T, Vilarino-Guell C, Soto-Ortolaza AI, Rajput A, et al. Genetic variants of alpha-synuclein are not associated with essential tremor. Movement disorders : official journal of the Movement Disorder Society. 2011;26(14):2552-6.
40. Zahorakova D, Ulmanova O, Kemlink D, Kofrankova M, Roth J, Martasek P, et al. No association with the ETM2 locus in Czech patients with familial essential tremor. Neuro endocrinology letters. 2010;31(4):549-52.
41. Zuo X, Jiang H, Guo JF, Yu RH, Sun QY, Hu L, et al. Screening for two SNPs of LINGO1 gene in patients with essential tremor or sporadic Parkinson's disease in Chinese population. Neuroscience letters. 2010;481(2):69-72.
42. Clark LN, Park N, Kisselev S, Rios E, Lee JH, Louis ED. Replication of the LINGO1 gene association with essential tremor in a North American population. European journal of human genetics : EJHG. 2010;18(7):838-43.

43. Vilarino-Guell C, Wider C, Ross OA, Jasinska-Myga B, Kachergus J, Cobb SA, et al. LINGO1 and LINGO2 variants are associated with essential tremor and Parkinson disease. Neurogenetics. 2010;11(4):401-8.
44. Thier S, Lorenz D, Nothnagel M, Stevanin G, Durr A, Nebel A, et al. LINGO1 polymorphisms are associated with essential tremor in Europeans. Movement disorders : official journal of the Movement Disorder Society. 2010;25(6):717-23.
45. Tan EK, Teo YY, Prakash KM, Li R, Lim HQ, Angeles D, et al. LINGO1 variant increases risk of familial essential tremor. Neurology. 2009;73(14):1161-2.
46. Clark LN, Kisselev S, Park N, Ross B, Verbitsky M, Rios E, et al. Mutations in the Parkinson's disease genes, Leucine Rich Repeat Kinase 2 (LRRK2) and Glucocerebrosidase (GBA), are not associated with essential tremor. Parkinsonism & related disorders. 2010;16(2):132-5.
47. Garcia-Martin E, Martinez C, Alonso-Navarro H, Benito-Leon J, Puertas I, Rubio L, et al. Paraoxonase 1 (PON1) polymorphisms and risk for essential tremor. European journal of neurology. 2010;17(6):879-81.
48. Keeling BH, Vilarino-Guell C, Soto-Ortolaza AI, Ross OA, Uitti RJ, Rajput A, et al. Histamine N-methyltransferase Thr105Ile is not associated with Parkinson's disease or essential tremor. Parkinsonism & related disorders. 2010;16(2):112-4.
49. Vilarino-Guell C, Ross OA, Wider C, Jasinska-Myga B, Cobb SA, Soto-Ortolaza AI, et al. LINGO1 rs9652490 is associated with essential tremor and Parkinson disease. Parkinsonism & related disorders. 2010;16(2):109-11.
50. Lorenz D, Klebe S, Stevanin G, Thier S, Nebel A, Feingold J, et al. Dopamine receptor D3 gene and essential tremor in large series of German, Danish and French patients. European journal of human genetics : EJHG. 2009;17(6):766-73.
51. Inashkina I, Radovica I, Smeltere L, Vitols E, Jankevics E. Case-control study of patients with essential tremor in Latvia. European journal of neurology. 2008;15(9):988-90.
52. Ledesma MC, Garcia-Martin E, Alonso-Navarro H, Martinez C, Jimenez-Jimenez FJ, Benito-Leon J, et al. The nonsynonymous Thr105Ile polymorphism of the histamine N-methyltransferase is associated to the risk of developing essential tremor. Neuromolecular medicine. 2008;10(4):356-61.
53. Tan EK, Lee J, Lim HQ, Yuen Y, Zhao Y. Essential tremor and the common LRRK2 G2385R variant. Parkinsonism & related disorders. 2008;14(7):569-71.
54. Vitale C, Gulli R, Ciotti P, Scaglione C, Bellone E, Avanzino L, et al. DRD3 Ser9Gly variant is not associated with essential tremor in a series of Italian patients. European journal of neurology. 2008;15(9):985-7.
55. Chen CM, Hou YT, Liu JY, Wu YR, Lin CH, Fung HC, et al. PPP2R2B CAG repeat length in the Han Chinese in Taiwan: Association analyses in neurological and psychiatric disorders and potential functional implications. American journal of medical genetics Part B, Neuropsychiatric genetics : the official publication of the International Society of Psychiatric Genetics. 2009;150b(1):124-9.
56. Blair MA, Ma S, Phibbs F, Fang JY, Cooper MK, Davis TL, et al. Reappraisal of the role of the DRD3 gene in essential tremor. Parkinsonism & related disorders. 2008;14(6):471-5.

57. Martinez C, Garcia-Martin E, Alonso-Navarro H, Benito-Leon J, Puertas I, Rubio L, et al. Glutathione-S-transferase P1 polymorphism and risk for essential tremor. European journal of neurology. 2008;15(3):234-8.
58. Martinez C, Garcia-Martin E, Alonso-Navarro H, Benito-Leon J, Puertas I, Rubio L, et al. Alcohol dehydrogenase 2 genotype and allelic variants are not associated with the risk for essential tremor. Clinical neuropharmacology. 2007;30(4):196-200.
59. Martinez C, Garcia-Martin E, Alonso-Navarro H, Jimenez-Jimenez FJ, Benito-Leon J, Garcia-Ferrer I, et al. Changes at the CYP2C locus and disruption of CYP2C8/9 linkage disequilibrium in patients with essential tremor. Neuromolecular medicine. 2007;9(2):195-204.
60. Tan EK, Prakash KM, Fook-Chong S, Yih Y, Chua E, Lum SY, et al. DRD3 variant and risk of essential tremor. Neurology. 2007;68(10):790-1.
61. Alonso-Navarro H, Martinez C, Garcia-Martin E, Benito-Leon J, Garcia-Ferrer I, Vazquez-Torres P, et al. CYP2C19 polymorphism and risk for essential tremor. European neurology. 2006;56(2):119-23.
62. Deng H, Xie WJ, Le WD, Huang MS, Jankovic J. Genetic analysis of the GABRA1 gene in patients with essential tremor. Neuroscience letters. 2006;401(1-2):16-9.
63. Lucotte G, Lagarde JP, Funalot B, Sokoloff P. Linkage with the Ser9Gly DRD3 polymorphism in essential tremor families. Clinical genetics. 2006;69(5):437-40.
64. Jeanneteau F, Funalot B, Jankovic J, Deng H, Lagarde JP, Lucotte G, et al. A functional variant of the dopamine D3 receptor is associated with risk and age-at-onset of essential tremor. Proceedings of the National Academy of Sciences of the United States of America. 2006;103(28):10753-8.
65. Deng H, Le WD, Guo Y, Huang MS, Xie WJ, Jankovic J. Extended study of A265G variant of HS1BP3 in essential tremor and Parkinson disease. Neurology. 2005;65(4):651-2.
66. Louis ED, Applegate L, Graziano JH, Parides M, Slavkovich V, Bhat HK. Interaction between blood lead concentration and delta-amino-levulinic acid dehydratase gene polymorphisms increases the odds of essential tremor. Movement disorders : official journal of the Movement Disorder Society. 2005;20(9):1170-7.
67. Sazci A, Ergul E, Bayulkem K. Association of the C677T and A1298C polymorphisms of methylenetetrahydrofolate reductase gene in patients with essential tremor in Turkey. Movement disorders : official journal of the Movement Disorder Society. 2004;19(12):1472-6.
68. Pigullo S, Di Maria E, Marchese R, Bellone E, Gulli R, Scaglione C, et al. Essential tremor is not associated with alpha-synuclein gene haplotypes. Movement disorders : official journal of the Movement Disorder Society. 2003;18(7):823-6.
69. Tan EK, Matsuura T, Nagamitsu S, Khajavi M, Jankovic J, Ashizawa T. Polymorphism of NACP-Rep1 in Parkinson's disease: an etiologic link with essential tremor? Neurology. 2000;54(5):1195-8.
70. Pigullo S, Di Maria E, Marchese R, Assini A, Bellone E, Scaglione C, et al. No evidence of association between CAG expansions and essential tremor in a large cohort of Italian patients. Journal of neural transmission (Vienna, Austria : 1996). 2001;108(3):297-304.
71. Agundez JA, Jimenez-Jimenez FJ, Tejeda R, Ledesma MC, Orti-Pareja M, Gasalla T, et al. CYP2D6 polymorphism is not associated with essential tremor. European neurology. 1997;38(2):99-104.

72. Stefansson H, Steinberg S, Petursson H, Gustafsson O, Gudjonsdottir IH, Jonsdottir GA, et al. Variant in the sequence of the LINGO1 gene confers risk of essential tremor. Nature genetics. 2009;41(3):277-9.
73. Thier S, Lorenz D, Nothnagel M, Poremba C, Papengut F, Appenzeller S, et al. Polymorphisms in the glial glutamate transporter SLC1A2 are associated with essential tremor. Neurology. 2012;79(3):243-8.
74. Muller SH, Girard SL, Hopfner F, Merner ND, Bourassa CV, Lorenz D, et al. Genome-wide association study in essential tremor identifies three new loci. Brain : a journal of neurology. 2016;139(Pt 12):3163-9.
